# Parent and caregiver preferences for eHealth programs

**DOI:** 10.1186/s12889-025-22612-8

**Published:** 2025-07-03

**Authors:** Nicole A. C. Tongol, Robert J. W. McHardy, Kaeley M. Simpson, Kailey E. Penner, Charlie Rioux, Emily E. Cameron, Ryan Giuliano, Lianne M. Tomfohr-Madsen, Ashley Stewart-Tufescu, Tracie O. Afifi, Tasmia Hai, Leslie E. Roos

**Affiliations:** 1 Department of Psychology, University of Manitoba, 190 Dysart Road, Winnipeg, MB R3T 2N2 Canada; 2 Department of Interdisciplinary Human Sciences, Texas Tech University, Lubbock, TX USA; 3 Department of Pediatrics and Child Health, University of Manitoba, Winnipeg, MB Canada; 4 Department of Educational and Counselling Psychology, and Special Education, University of British Columbia, Vancouver, BC Canada; 5 Children’s Hospital Research Institute of Manitoba, University of Manitoba, Winnipeg, MB Canada; 6 Faculty of Social Work, University of Manitoba, Winnipeg, MB Canada; 7 Department of Community Health Sciences, University of Manitoba, Winnipeg, MB Canada; 8 Department of Educational & Counselling Psychology, McGill University, Montreal, QC Canada; 9 Faculty of Education, Simon Fraser University, Burnaby, BC Canada

**Keywords:** User preferences, Parenting programs, Young children, Online resources, Families, Mental health

## Abstract

**Background:**

Online programs serve as an important avenue for delivering mental health and parenting services worldwide. The quantity of online programs proliferated during the COVID-19 pandemic with developers emphasizing the potential to improve accessibility and reduce barriers of in-person programming (e.g., arranging transportation, childcare, and scheduling). However, Canadian parents’ and caregivers’ preferences for features they desire in online family mental health supports are unknown. Understanding these preferences would better allow for the creation of programs that are best suited to meet parents’ needs. Thus, the present study examined parent mental health program preferences, barriers to access, and how different sociodemographic factors predicted preferences for aspects such as program features (e.g., duration delivery format).

**Methods:**

Self-report surveys were administered in 2023 via the online crowdsourcing platform AskingCanadians to parents and primary caregivers of children ages 0 to 5 years. Descriptive statistics examined parent mental health program preferences and barriers. Regression models examined sociodemographic predictors of these preferences and barriers.

**Results:**

Participants identified a range of preferences across program structure and coaching, as well as challenges associated with program access. Parents most preferred programs with a web-based delivery format (72%), a duration of 2–4 weeks (27%), and psychologists as program coaches (51.4%). The most highly endorsed barriers were lack of time (42.2%) and limited internet access (25.1%). Sociodemographic factors including parent gender, household income, education, and ethnicity also consistently predicted preference for various program characteristics.

**Conclusions:**

This research provides an important first step toward creating more accessible online mental health and parent mental health programs by ensuring the voices of the parents who will use these services are heard in program development and adaptation. Future research should investigate how to address accessibility and inclusivity barriers to participating in parent mental health programs for diverse families based on their differential preferences.

**Supplementary Information:**

The online version contains supplementary material available at 10.1186/s12889-025-22612-8.

## Introduction

Family-focused programs and resources are crucial supports for families, but waitlists for in-person mental health and parenting services are characteristically long [1]. Almost 50% of Canadians must wait about one month for community mental health counselling, while wait times for seeing a psychiatrist are 2.5 weeks for urgent patients and 15.5 weeks for non-urgent patients [2, 3]. In the United States, median wait times are just over one month for telepsychiatry, in contrast to over two months for in-person psychiatry appointments [4]. In both Australia and the United Kingdom, it was found that a quarter to almost two-thirds of patients wait more than 12 weeks for in-person mental health care services [5, 6]. Evidently, there is a crisis in many countries regarding wait times for accessing mental health care, in which people’s challenges and symptoms may worsen while they wait for treatment.

While many Canadians suffered because of the pandemic, parents (inclusive of all primary caregivers of children such as biological parents, adoptive parents, foster parents, kinship parents) who supported and lived with their children seemed to be a population that was in particular distress [7]. The closure of daycares, concerns about their children becoming sick, and the need to work from home while simultaneously caring for their children caused parents increased stress and mental health difficulties [7, 8]. Parents reported increased psychological distress, higher alcohol consumption, greater concerns about staying safe from domestic violence, challenges with work-family balance, and more negative parent-child interactions [7–9]. Thus, it is evident that there is a significant and crucial need to support parent mental health in ways that are easily accessible, inclusive, and adaptable to the various needs of different populations of parents.

Online programs provide an avenue for addressing the crucial needs of parents in more accessible ways while families remain on waitlists for in-person services. For example, parents might use an online program to learn parenting skills for challenging child behaviours. They may also try an app-based program that requires less direct time face-to-face with a clinician. This would not replace in-person services but provide families with support while they wait to receive in-person services. While online mental health and parenting programs were available before the COVID-19 pandemic, they proliferated after quarantine protocols were put into place and the transition to virtual services became a necessity for many families and service providers [10]. This transition was urgent and done rapidly, making it difficult to utilize best practices in program development, adaptation, and delivery.

Best-practice guidelines released by the American Telemedicine Association (ATA) and the American Psychiatric Association (APA) suggest that service providers conduct a telehealth needs assessment (detailing things like staffing and training needs, an evaluation plan, and sustainability) in developing programs [11]. However, several parent mental health programs have been developed for parents despite limited research to date having examined what parents want from these online programs [12–15]. Therefore, the present study sought to better understand the needs and preferences of Canadian parents for online parent mental health programming.

### Online parenting programs and resources

For the purposes of this study, parent mental health programs are defined as services that aim to help parents with family mental health challenges (e.g., managing depression or anxiety for both the parents and their children), teach child emotional regulation skills, and manage difficult child behaviours. These programs are typically longer-term interventions, classes, or activities with a predefined curriculum and are often facilitated by experts with specific certifications (e.g., mental health professionals, such as psychologists, or co-facilitation by parents and community members with relevant lived experiences). There are several newer parent mental health programs (e.g., online mindful parenting programs, virtual parenting interventions for children with neurological conditions) that were individually evaluated for their efficacy and perceived benefits [12–17]. However, it is still unclear what specific facets of parent mental health programs, such as program length or delivery format, are desired by parents.

Parents may access parent mental health programs online for many reasons (e.g., building new parents’ confidence and parenting efficacy, gaining parenting information, reducing stress) and through various modalities (e.g., mobile applications, websites, social media) [13, 18, 19]. The literature on parent mental health programs mostly consists of individual program evaluation instead of larger program reviews [12, 14, 15, 17, 20–25]. Individual program evaluations might focus more on whether the program is working as intended (e.g., lowering depressive symptoms, increasing parenting efficacy), whereas a program review might focus on efficacy as well as feasibility, acceptability, and what aspects of the program parents enjoyed or see room for improvement. Systematic reviews and meta-analyses on online parenting interventions from before the COVID-19 pandemic have found overall positive effects for improving outcomes like parenting knowledge, confidence, and parenting/child behaviours, while reducing parenting stress and negative parent-child interactions [26, 27]. For example, researchers found overall positive effects of online interventions for improving parent mental health symptoms in a systematic review and meta-analysis for studies completed before the pandemic [28]. Still, very few studies directly investigate what parents view as important in these programs. Researchers examined parent preferences for connecting with peers in eHealth programming, but further research is needed to investigate parent preferences with a broader scope and with an emphasis on mental health needs unique to this population [29].

### Why do we need to know the preferences of parents?

Consideration of diverse voices is crucial when seeking to understand Canadian parents’ program preferences. While both in-person and online parent mental health programs may improve family outcomes, these supports have not been evaluated across representatively diverse parent populations [26, 30]. To be most relatable and inclusive, parent mental health programs should have a wide reach, be adaptable across parent groups, and be informed by the thoughts and experiences of the people who will use them. Co-development of programs and participatory action research have the potential to allow researchers and service providers to create parent mental health programs that follow best practices, are evidence-informed, and have the best chance at being relevant. Studies with parents and families have also demonstrated success with participatory action research [31, 32]. Thus, a good first step in intervention development is to understand the perspectives of the population that the program is intending to serve.

#### Preferences based on parent sociodemographic factors

While some studies broadly examine whether parent participants find programs acceptable, few of these studies examined which specific facets (e.g., length, content) of parent mental health programs participants prefer [15, 22, 24, 31]. Even still, the primary aim of these studies was to understand how participants approved or disapproved of the specific program being administered as opposed to understanding broader preferences [18, 21, 22]. Additionally, few studies have investigated preferences for parent mental health programs based on parent sociodemographic characteristics. Although some studies have found associations between specific parent sociodemographic characteristics (e.g., age, gender, household income) and program features, most were conducted prior to COVID-19 and outside of the Canadian context. For example, parents who are younger in age and parents with young children seem to more frequently engage in accessing supports, but this pattern is likely due to several factors not yet explored, such as socioeconomic status, eHealth literacy, accessibility of local programs, and employment [18, 19, 33]. In another study, parents who identified as a woman preferred accessing parenting resources through social media [18]. Parents with fewer resources (i.e., low socioeconomic status, low household income, unemployment difficulties) show reduced engagement with in-person programs. Instead, they may prefer engagement through social media or parenting apps, due to the lower demand in terms of parent participation [18, 19, 25, 34].

#### Barriers to accessing parenting supports

Broadly, many factors prevent parents from easily accessing parenting supports. Some barriers relate to the capacity and needs of parents who may already be overwhelmed from parenting challenges and navigating confusing mental healthcare systems. For example, with in-person programs, barriers may be factors such as lack of childcare, transportation/commuting difficulties, or having to accommodate a busy work schedule. However, barriers specific to an online program may be limited access to high-speed internet. Some barriers may also be relevant to both in-person and online programs, such as household income, lack of time, and scheduling challenges [21, 24, 30, 35, 36]. Thus, some barriers may be the same for both modes of delivery, but some may be different, further showcasing why it is important to assess for barriers to program access. One study also identified that barriers were more difficult to manage for parents who were lower income and ethnically diverse [24]. Other barriers may be systemic, such as long waitlists, lack of services, and stigma related to mental health diagnoses [30, 37]. Some barriers may also have to do with identity matching. For example, some individuals might prefer or feel more comfortable with a therapist or coach who matches their ethnic or gender identity. However, studies looking at matching age, gender, and ethnic identities between therapists and participants tend to show inconclusive results [38–40]. Additional research is needed to better understand what makes a program accessible and acceptable, which may facilitate the development of parent mental health programs that are more accessible, understandable, and considerate of individual needs and circumstances.

### The present study

To date, few research studies have investigated parent preferences for online parent mental health programs. Little is known about parents’ perceived usefulness of these online programs, the characteristics they look for within a program, preferences for formats of receiving material, and program duration preferences. Therefore, we sought to answer the following research questions: (Q1) what are the eHealth program preferences of parents, and (Q2) which sociodemographic factors predict these preferences? Specific preferences examined include (1) program features, (2) program length, (3) program structure, (4) program coach credentials, (5) content delivery medium, (6) barriers to program access, (7) peer shared identities, and (8) coach shared identities. “Program features” refers to items that parents might want included in an online program, including things like whether a coach or facilitator is present. “Program structure” refers to how parents would want the program to be organized (i.e., in a self-directed way or in a way structured by the developers). “Shared identities” refers to sharing certain identities in common with others (e.g., sharing the same age, gender, or ethnicity in common with a peer).

The present study is informed and guided by strategies for patient-oriented research (SPOR) by the Canadian Institute of Health Research (CIHR) [41]. The present research was not funded by CIHR. However, one goal of this study is to act as an important first step towards future research that will aim to fully engage parents and their families in the development of parent mental health programs. In supporting parent mental health, children are also supported through breaking the intergenerational transmission of poor mental health.

## Method

### Participants

Data were collected from June-July 2023 with approval from the Research Ethics Board at the University of Manitoba (HE2023-0124). A total of 606 participants were recruited and took an online survey on AskingCanadians to facilitate recruitment of a sample from across Canada for this research using crowdsourcing techniques. AskingCanadians is a strong platform, ensuring that their panel of participants is authentic and consistent as part of their standard procedure, removing non-participative respondents from the panel every six months, and collecting participant feedback to improve their services [42]. Participants were eligible if they currently resided in Canada, were at least 18 years old, identified as a parent to a child 0–5 years old, were comfortable understanding, reading, and writing in English, and had access to an electronic device with internet access (e.g., phone or computer).

### Procedure

Participants who had an existing profile on the AskingCanadians platform and matched with our eligibility criteria were sent an invite to complete the study survey. Interested participants clicked a link that redirected them to the consent form on the University of Manitoba’s secure Research Electronic Data Capture (REDCap) server [43, 44]. If participants consented to the survey, they were redirected to a brief 1–2-minute eligibility screener. If deemed eligible, participants were prompted to complete the full 25–30-minute survey. Upon completing the survey, participants were compensated with loyalty program points equivalent to $9.00 CAD through AskingCanadians which could be redeemed through programs of their choice (e.g., Hudson’s Bay Rewards, Petro-Points). Incomplete surveys were still analyzed, and participants were informed that they could withdraw, decline to participate, or stop participating at any time without consequence.

### Measures and materials

Participants completed the following measures in the order outlined below.

#### Sociodemographic questions

Sociodemographic factors examined included parent age, gender, household income, financial well-being, employment, education, marital status (married/common-law, unmarried), ethnicity (White, non-White), first-language spoken at home (English, not English), number of children in the household, and whether or not parents were a first-time parent). Questions were considered based on variables of interest and the Canadian census [45].

#### Parent preferences questionnaire

This 30-item measure was created by our research team to evaluate specific parent preferences for parent mental health programs or resources (see supplementary materials for full instrument). Multiple members of our research team are experienced in delivering online interventions to parents and families, having heard feedback for how they can be improved [16, 17]. Therefore, the measure was not directly informed by parent participants, but it was created using the research team’s expertise on family experiences in online parent mental health programs. Example questions include items such as “Content through eHealth resources can be delivered in a variety of different ways. How would you want to access content through a program? Check all that apply,” with multiple choice possible answers including items like, “web-based portal on computer or laptop,” “application on phone,” “teletherapy on videoconferencing platforms,” etc. Other questions asked about program length, specific desirable eHealth features (e.g., content developed by experts, supportive online forums), program facilitation (e.g., having a self-directed or structured program), what information should be included (e.g., specific and tailored versus a library of information), who to receive coaching from (e.g., peers, psychologists, social workers), and how to present content (e.g., audio-only, audio-visual). The final question asked about barriers to accessing eHealth programs specifically.

#### Barriers and facilitators questionnaire

This four-item measure was created by the researchers to understand what parents perceive as preventing and encouraging for them to access in-person and online family mental health supports. Questions included multiple choice options with opportunities for providing further information via open text boxes. Questions and response items were developed through a review of the literature and the authors’ own experiences as parents and clinicians, including years of research with parent advisory boards across other projects [46–51]. Authors reviewed the literature on barriers and facilitators for online mental health programs, including those cited in this manuscript [34–36]. They also reflected on their experiences as parents and clinicians, including what clients have detailed regarding frustration with the mental health care system and its resources. These frustrations were echoed by parents in advisory boards across other projects. This measure was reviewed and edited by multiple members of the research team.

### Data analytic plan

All analyses, unless otherwise specified, were conducted in Statistical Package for Social Science (SPSS) software 29.0.1.0 [52]. Descriptive statistics of parent mental health program preferences were examined to address Research Question 1, seeking to characterize average parent eHealth program preferences.

Research Question 2 sought to identify parent sociodemographic variables that predict parent eHealth program preferences. Prior to conducting these analyses, patterns of missingness were examined. Study outcomes were 9.42% missing, with highest missingness (~ 10–20%) among program length, structure, and shared identity outcomes. A total of 65.18% cases were complete. Little’s MCAR revealed data were not missing completely at random (χ^2^(3066) = 3239.40, *p* =.015) [53]. Higher missingness was predicted by gender, household income, financial well-being, employment status, education, and various program preferences (*p*’s < 0.05). Multiple imputation with fully conditional specification used five imputations to generate estimates for missing values across all study variables [54]. Sensitivity analyses were run with both imputed values and unimputed values to examine robustness of results. Results pertaining to research question 2 are reported with imputations/imputed values. Any differences are highlighted in the results tables at the end of the document. Further, results without imputation are presented in supplemental materials. Following multiple imputation, regression models were completed. Sociodemographic predictors were selected for each Research Question 2 sub-question if they were significantly associated with any outcome in the question. For variable selection, α = 0.05 represented a significant zero-order correlation (if on a ratio scale) or significant *F*-tests or *t*-tests (for categorical variables). Due to the exploratory nature of the research questions, decisions about which predictors to include in each regression model were data driven.

Whether a given model used linear, binary logistic, or Poisson regression depended on whether the outcome of interest was on a ratio, binary, or count scale, respectively. Regression assumptions were examined for linear, binary logistic, and Poisson regression models. For linear regression, outliers were assessed by manually examining standardized residuals. No outliers (i.e., standardized residuals of *SD* > 3.00) were identified. For binary logistic regression, the Box-Tidwell procedure examined linearity between each predictor and logit [55]. For Poisson regression, each model’s dispersion coefficient determined whether the data followed a Poisson distribution [56]. Because all Poisson models were over-dispersed (as is typical in empirical research), these data were instead modelled with negative binomial regression to handle over-dispersion [56, 57]. All negative binomial regression models were also run in the form of hurdle regression models using the R language to ensure robustness and manage zero-inflation [57, 58]. Multicollinearity was examined for all models using Variance Inflation Factor (VIF) and eigenvalue Condition Index (CI) collinearity diagnostics in SPSS [59, 60]. All regression models had VIF < 2.55, suggesting no concern of collinearity [59, 60].

This study models 238 regression coefficients in total. At α = 0.05, the family-wise error rate was > 99.9%; representing a high probability that one or more effects are false-positives. Significant effects were instead determined using the Benjamini-Hochberg procedure, a method that controls the false-positive rate by computing a critical value for each effect [61, 62]. Only effects that meet their critical value, as defined by the Benjamini-Hochberg procedure, are reported in these results. Further, these significant effects are clearly marked in tables.

## Results

Key patterns across research questions are outlined below, with additional findings in tables at the end of the document and within the supplementary materials.

### Description of sample

Unimputed means, standard deviations, and ranges are presented for all participant sociodemographic variables in the supplementary materials. On average, participants were 33.88 years old (*SD*_age_ = 6.87) and had just under two children (*M* = 1.73, *SD* = 0.91). Most participants identified as women (63.4%), were married or common-law (79.2%), were employed full-time (61.9%), and spoke English as their primary language at home (83.8%). The median respondent held a bachelor’s degree, had a household income between $70,000 and $99,999 per year, and reported their perceived financial position to be “Average.” Almost half of the participants lived in Ontario (48.0%; *n* = 291), with the next largest groups of participants coming from Alberta (15.7%; *n* = 95), British Columbia (12.9%; *n* = 78), and Québec (8.3%; *n* = 50). The remaining 92 respondents reported living in other parts of Canada, with all Canadian provinces and territories being represented except for Nunavut and the Northwest Territories. Thus, based on the Canadian census, this sample is relatively representative of the Canadian population [45, 63, 64].

Participants were free to select as many different ethnic background categories as applied to them. A slight majority of participants (54.9%; *n* = 331) identified as only being White while the remainder (*n* = 272) reported a range of ethnic identities across South Asian, East Asian, Indigenous, and Latin American, Black African, Southeast Asian, Black Caribbean, Middle Eastern, Black Canadian, and Indo-Caribbean. Three participants declined to answer. Most survey participants were born in Canada (74.1%; *n* = 449).

Several parental sociodemographic characteristics were associated with each other before the multiple imputation procedure (table in supplementary materials). At the α = 0.005 level, being an older parent was positively associated with household income, higher levels of education, and having more children. Being an older parent was negatively associated with perceived financial well-being and being a first-time parent (i.e., older parents were less likely to be first-time parents). Parents who identified as a woman reported having a lower household income and lower perceived financial well-being. Having a higher household income was linked with higher perceived financial well-being, being married or common-law, and having a higher education level. Having a higher perceived financial well-being was similarly positively correlated with education level and being married or common-law. Higher education level was negatively linked with being White, primarily speaking English at home, and number of children, but was positively linked with being married and being a first-time parent. Being employed full-time was associated with lower levels of education. Finally, identifying as White was linked with speaking primarily English at home.

### Research question 1: characterizing parent eHealth program preferences

Looking to average survey responses before multiple imputation shows general trends in Canadian parent preferences for eHealth programs (table in supplementary materials). Specific eHealth program preferences examined include (1) program features, (2) program length, (3) program structure, (4) program coach credentials, (5) content delivery medium, (6) barriers to program access, (7) peer shared identities, and (8) coach shared identities.

When respondents were asked to check all acceptable (1) program features, the most popular choice appeared to be content delivered via web-based formats (*M*_checked_ = 0.97; *SD* = 0.92), followed by app (*M*_checked_ = 0.72; *SD* = 0.91), coach (*M*_checked_ = 0.70; *SD* = 1.01), and teletherapy (*M*_checked_ = 0.26; *SD* = 0.54). The most preferred (2) program length was reported to be 2–4 weeks (27.3%; *n* = 132), followed by 1–2 months (24.8%; *n* = 120). In terms of (3) program structure, parents appeared mixed on whether they wanted programs to be self-directed/guided, peer-driven/expert-driven, asynchronous/synchronous, and have tailored/open content. When asked to rate these preferences on a scale from 0 to 100, the average response fell near the middle with healthy variation (range = 43.69–54.75).

When asked about preferences for (4) program coach credentials, parents reported psychologist coaches as the most popular (51.4% endorsed), followed by medical doctor (40.5%), social worker (33.9%), community member (26.4%), and peer coaches (22.6%). In terms of (5) content delivery medium, almost half of participants reported a preference for program content with both audio and visual components (48.1%). When asked about potential (6) barriers to program access, the average participant endorsed almost two of eight possible barriers (*M* = 1.59; *SD* = 1.48). The most endorsed barrier was lack of time (42.2% of respondents), followed by limited internet access (25.1%) and lack of childcare (21.4%). Finally, when asked to rank the importance of (7, 8) program peers and coaches sharing aspects of their identity (e.g., age, gender, cultural identity), respondents appeared mixed. Average responses to most items fell in the middle of “Very unimportant” (1) to “Very important” (5), with no responses falling closer to “Very unimportant” than “Very important” (Range = 3.04–3.68).

While average program preferences are informative, it is important to disaggregate program preferences by important sociodemographic characteristics given the heterogeneity of the sample.

### Research question 2: sociodemographic predictors of parent eHealth program preferences

Parent sociodemographic factors were next examined as predictors of reported eHealth program preferences. Specific eHealth program preferences examined aligned with Research Question 1 to include (1) program features, (2) program length, (3) program structure, (4) program coach credentials, (5) content delivery medium, (6) barriers to program access, (7) peer shared identities, and (8) coach shared identities.

#### Which sociodemographic factors predict parent preferences for program features?

**Fig. 1:**
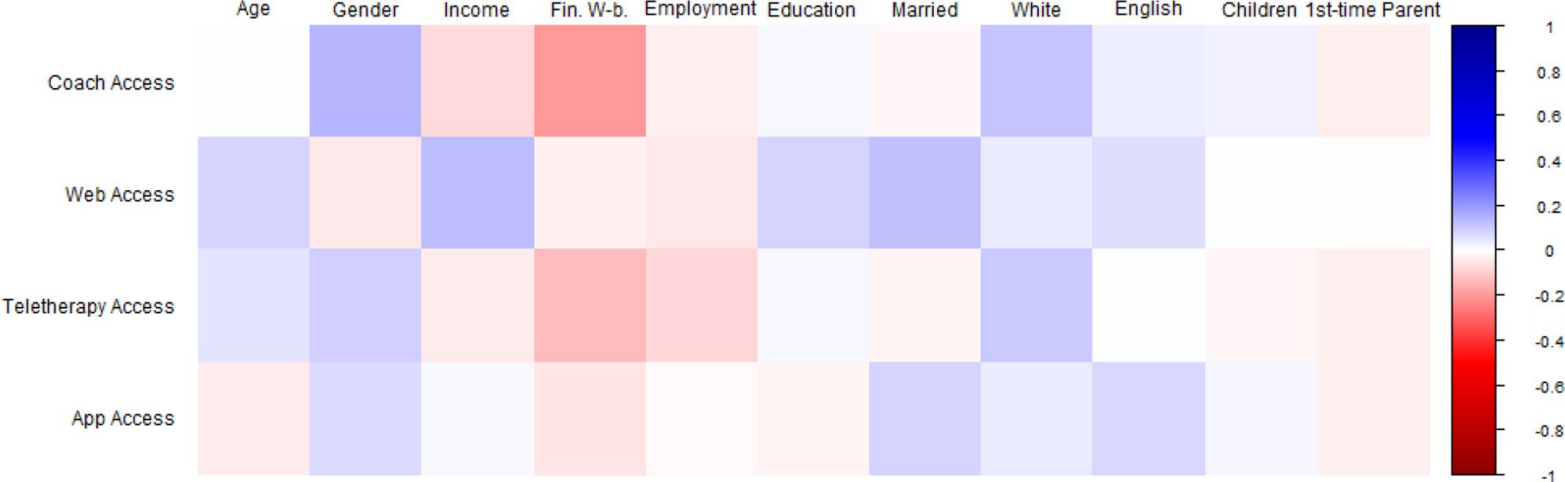
Bivariate correlations between sociodemographic factors and program feature preferences. *Note.* Square *color* represents correlation *directionality* (blue = positive, red = negative). Square color *darkness* represents correlation *strength* (darkest blue is *r* = 1.00, darkest red is *r* = -1.00, White is *r* =.00). Sociodemographic variables at top, program preferences at left. At top: ‘Age’ = Parent age in years; ‘Gender’ = Dichotomized Gender (Ref: Man); ‘Income’ = Household Income; ‘Fin. W-b.’ = Financial Well-being; ‘Employment’ = Dichotomized Employment (Ref: Not Working); ‘Education’ = Education level; ‘Married’ = Dichotomized Marital Status (Ref: Not Married); ‘White’ = Dichotomized Ethnicity (Ref: Not White); ‘English’ = Dichotomized English as Home Language (Ref: No); ‘Children’ = Number of Children; ‘1st-time Parent’ = Dichotomized First-time Parent (Ref: No)

We first examined which sociodemographic factors might predict parent preferences for eHealth program features, including web-based program access, app-based program access, program coaches, and teletherapy options (see Fig. 1 for correlational relationships). Identifying as a woman as well as greater household income, lower perceived financial well-being, greater education level, being married, and identifying as White were all significantly associated with endorsing more program features. Negative binomial regression examining parent preferences for program coaches was significant (χ^2^(9) = 35.901, *p* <.001). This model (Table 1a) found that each increase in financial well-being was associated with less frequent endorsement of program coaches (Exp(*B*) = 0.774, 95% CI [0.678, 0.883], *p* <.001). Additional models examining web-based (χ^2^(9) = 24.612, *p* =.003; Table 1b) and teletherapy-based access options (χ^2^(11) = 20.800, *p* =.014; Table 1c) were also significant, but did not evidence significant effects. The model examining preferences for app-based access was not significant overall, *p*’s > 0.05 (see Table 1d).

**Table 1:** Negative binomial regression model summary for sociodemographic predictors of program feature preferences (RQ 1)

Outcome	(a) Program Coach Access			(b) Web-based Access			(c) Teletherapy Access			(d) App-based Access		
	Exp(B)	95% CI	*p*	Exp(B)	95% CI	*p*	Exp(B)	95% CI	*p*	Exp(B)	95% CI	*p*
Intercept	0.48	[0.17, 1.31]	0.149	1.08	[0.64, 1.81]	0.771	0.08	[0.01, 0.63]	0.017	0.60	[0.32, 1.15]	0.126
Gender Identity	1.26	[0.97, 1.65]	0.087	0.91	[0.76, 1.09]	0.306	1.23	[0.83, 1.82]	0.297	1.18	[0.95, 1.47]	0.130
Household Income	1.00	[0.93, 1.07]	0.972	1.06	[1.01, 1.11]	0.022	1.01	[0.91, 1.12]	0.871	1.02	[0.97, 1.09]	0.415
Financial Well-being	0.77	[0.68, 0.88]	< 0.001*	0.90	[0.82, 0.99]	0.034	0.79	[0.65, 0.96]	0.017	0.94	[0.84, 1.06]	0.310
Education (Ref: < High school)	-	-	-	-	-	-	-	-	-	-	-	-
Graduate degree	2.58	[0.92, 7.22]	0.071	0.90	[0.56, 1.45]	0.669	6.18	[0.81, 47.0]	0.078	0.87	[0.47, 1.59]	0.641
Bachelor’s	2.32	[0.87, 6.20]	0.092	0.83	[0.53, 1.30]	0.412	4.78	[0.66, 34.7]	0.121	0.83	[0.47, 1.47]	0.519
College	2.08	[0.78, 5.56]	0.141	0.72	[0.45, 1.13]	0.155	5.41	[0.75, 39.3]	0.094	0.84	[0.48, 1.46]	0.535
High school	2.33	[0.89, 6.14]	0.085	0.78	[0.49, 1.23]	0.280	4.85	[0.67, 34.9]	0.116	1.06	[0.61, 1.83]	0.839
Married	1.04	[0.76, 1.43]	0.814	1.27	[1.00, 1.62]	0.047	0.93	[0.62, 1.41]	0.743	1.38	[1.05, 1.81]	0.021
White	1.32	[1.03, 1.69]	0.028	1.08	[0.91, 1.28]	0.378	1.40	[0.98, 2.00]	0.067	1.04	[0.84, 1.28]	0.712

Note. ^*^ = *p*-value less than the Benjamini-Hochberg critical *p*-value for this effect. ^ζ^ = Effect significance differs from the supplementary model without multiple imputation. “Gender Identity” is a variable dichotomized ‘woman’ (1) or ‘man’ (0). “Married” and “White” are all variables dichotomized ‘Yes’ (1) or ‘No’ (0)

#### Which sociodemographic factors predict parent preferences for program length?

**Fig. 2:**
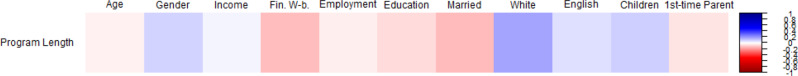
Bivariate correlations between sociodemographic factors and program length preferences. Note. Square color represents correlation directionality (blue = positive, red = negative). Square color darkness represents correlation strength (darkest blue is *r* = 1.00, darkest red is *r* = -1.00, White is *r* =.00). Sociodemographic variables at top, Parent preferences at left. At top: ‘Age’ = Parent age in years; ‘Gender’ = Dichotomized Gender (Ref: Man); ‘Income’ = Household Income; ‘Fin. W-b.’ = Financial Well-being; ‘Employment’ = Dichotomized Employment (Ref: Not Working); ‘Education’ = Education level; ‘Married’ = Dichotomized Marital Status (Ref: Not Married); ‘White’ = Dichotomized Ethnicity (Ref: Not White); ‘English’ = Dichotomized English as Home Language (Ref: No); ‘Children’ = Number of Children; ‘1st-time Parent’ = Dichotomized First-time Parent (Ref: No)

We next examined which sociodemographic factors might predict parent preferences for program length. Lower perceived financial well-being, being unmarried, identifying as non-White, and having fewer children all significantly correlated with preference for greater program length (see Fig. 2 for correlational relationships). A linear regression examining parent preferences for program length was significant (*F*(5, 600) = 5.742, *p* <.001, *R*^2^ = 0.046). This model (Table 2) showed that longer programs were preferred by those who identified as White (β = 0.140, *t* = 3.280, *p* =.001).

**Table 2:** Linear regression model summary for sociodemographic predictors of program length preferences (RQ 2)

Outcome	Program Length			
	B	β	t	*p*
Intercept	3.23		9.48	< 0.001
Household Income	0.07	0.09	1.99	0.046
Financial Well-being (ζ)	-0.19	-0.12	-2.24	.033^ζ^
Married (ζ)	-0.49	-0.12	-2.44	.020^ζ^
White	0.45	0.14	3.28	0.001*
Number of Children	0.16	0.09	1.98	0.050

Note. ^*^ = *p*-value less than the Benjamini-Hochberg critical *p*-value for this effect. ^ζ^ = Effect significance differs from the supplementary model without multiple imputation. “Married” and “White,” are variables dichotomized ‘Yes’ (1) or ‘No’ (0)

#### Which sociodemographic factors predict parent preferences for program structure?

**Fig. 3:**
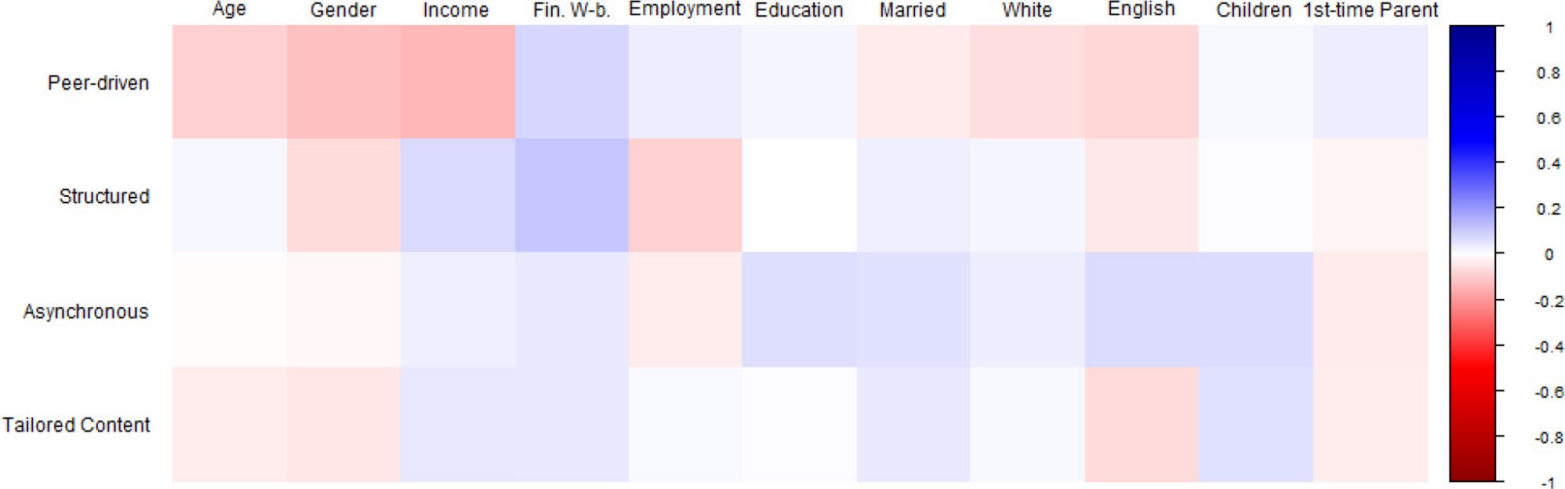
Bivariate correlations between sociodemographic factors and program structure preferences. Note. Square color represents correlation directionality (blue = positive, red = negative). Square color darkness represents correlation strength (darkest blue is *r* = 1.00, darkest red is *r* = -1.00, White is *r* =.00). Sociodemographic variables at top, Parent preferences at left. At top: ‘Age’ = Parent age in years; ‘Gender’ = Dichotomized Gender (Ref: Man); ‘Income’ = Household Income; ‘Fin. W-b.’ = Financial Well-being; ‘Employment’ = Dichotomized Employment (Ref: Not Working); ‘Education’ = Education level; ‘Married’ = Dichotomized Marital Status (Ref: Not Married); ‘White’ = Dichotomized Ethnicity (Ref: Not White); ‘English’ = Dichotomized English as Home Language (Ref: No); ‘Children’ = Number of Children; ‘1st-time Parent’ = Dichotomized First-time Parent (Ref: No)

We next examined which sociodemographic factors predict parent preferences for (a) peer-driven versus expert-driven and (b) self-directed versus structured programs (see Fig. 3 for correlational relationships). Younger parent age, identifying as a man, lower household income, higher perceived financial well-being, and lower employment were significantly associated with preferences for peer-driven and structured programs. No sociodemographic characteristics were associated with asynchronous or tailored content preferences at the bivariate level. Linear regression examining parent preferences for a peer- versus expert-driven program structure was significant (F(6, 599) = 5.070, *p* <.001, R^2^ = 0.048). This model (Table 3a) showed that expert-driven program structure was preferred by parents with higher household income (β = − 0.189, t = -3.947, *p* <.001). The model examining preferences for self-directed versus structured programs was significant overall (F(6, 599) = 3.163, *p* =.005, R^2^ = 0.031). This model (Table 3b) showed that structured programs were preferred by stay-at-home parents relative to working parents (β = − 0.149, t = -2.963, *p* =.004). Additional models examined preferences for asynchronous versus synchronous and tailored versus broad program content, but neither model omnibus was significant, p’s > 0.05 (see Tables 3c, 3d).

**Table 3:** Linear regression model summary for sociodemographic predictors of program structure preferences (RQ 3)

Outcome	(a) Peer- (vs. Expert-driven)				(b) Self-directed (vs. Structured)				(c) Asynchronous (vs. Sync.)				(d) Tailored (vs. Broad) Content		
	B	β	t	*p*	B	β	t	*p*	B	β	t	*p*	Exp(B)	95% CI	*p*
Intercept	52.79		7.17	< 0.001	45.23		6.37	< 0.001	50.93		6.19	< 0.001	0.82		0.778
Age	-0.12	− 0.04	-0.77	0.444	0.09	0.03	0.63	0.527	-0.01	− 0.00	-0.05	0.962	0.99	[0.96, 1.02]	0.408
Gender Identity (ζ)	-5.76	− 0.12	-2.61	.009^ζ^	0.27	0.01	0.12	0.903	0.57	0.01	0.25	0.807	0.86	[0.55, 1.36]	0.518
Household Income	-2.25	− 0.19	-3.95	< 0.001*	-0.04	− 0.00	-0.06	0.953	0.23	0.02	0.39	0.699	1.03	[0.93, 1.13]	0.601
Financial Well-being	2.26	0.10	1.92	0.057	1.23	0.06	1.12	0.264	0.90	0.04	0.79	0.431	1.05	[0.86, 1.28]	0.639
Employment (Ref: Not working)	-	-	-	-	-	-	-	-	-	-	-	-	-	-	-
Stay-at-home	2.99	0.05	0.61	0.544	-4.70	− 0.08	-1.21	0.225	-2.63	− 0.04	-0.63	0.526	1.06	[0.51, 2.21]	0.880
Working	1.61	0.03	0.39	0.699	4.53	0.09	1.25	0.213	-2.25	− 0.04	-0.65	0.519	1.02	[0.56, 1.86]	0.943
Employment (Ref: Working)	-	-	-	-	-	-	-	-	-	-	-	-	-	-	-
Stay-at-home	1.38	0.02	0.43	0.671	-9.23	− 0.15	-2.96	0.004*	-0.37	− 0.01	− 0.11	0.912	1.04	[0.59, 1.81]	0.902
Not working	-1.61	− 0.02	-0.39	0.699	-4.53	− 0.06	-1.25	0.213	2.25	0.03	0.65	0.519	0.98	[0.54, 1.78]	0.943

Note. ^*^ = *p*-value less than the Benjamini-Hochberg critical *p*-value for this effect. ^ζ^ = Effect significance differs from the supplementary model without multiple imputation. “Gender Identity” is a variable dichotomized ‘woman’ (1) or ‘man’ (0)

#### Which sociodemographic factors predict parent preferences for program coach credentials?

**Fig. 4:**
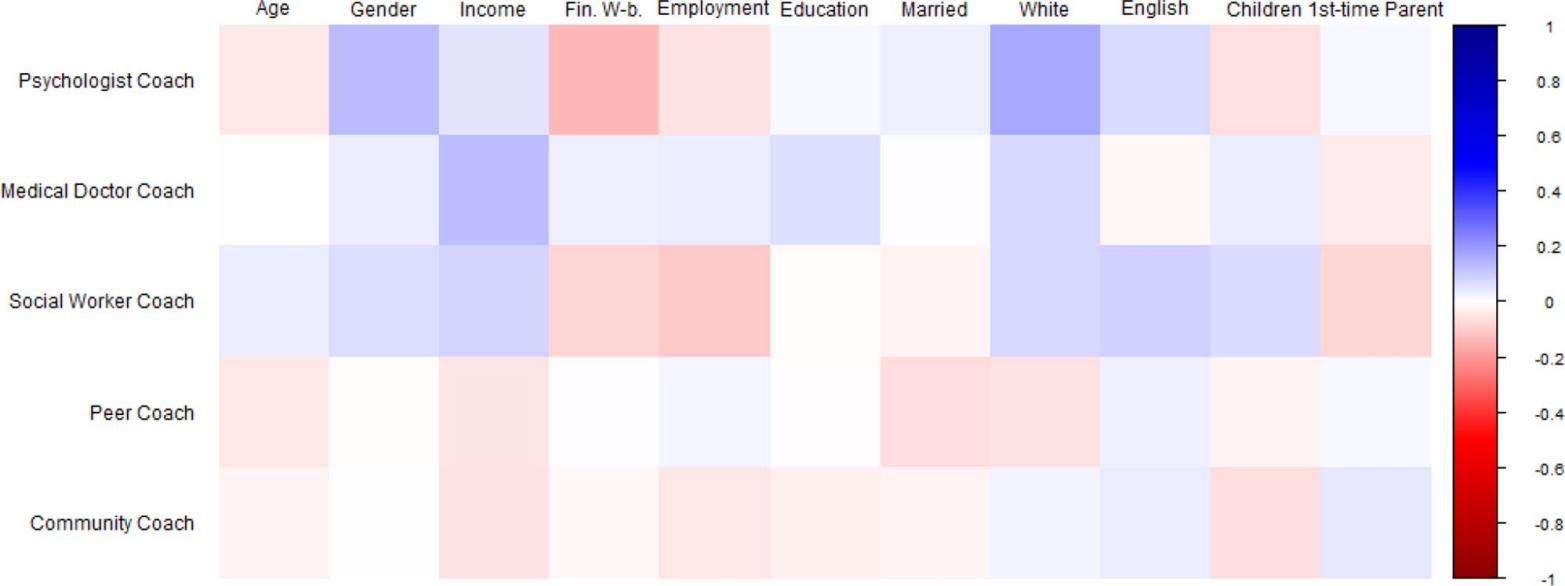
Bivariate correlations between sociodemographic factors and program coach preferences. Note. Square color represents correlation directionality (blue = positive, red = negative). Square color darkness represents correlation strength (darkest blue is *r* = 1.00, darkest red is *r* = -1.00, White is *r* =.00). Sociodemographic variables at top, Parent preferences at left. At top: ‘Age’ = Parent age in years; ‘Gender’ = Dichotomized Gender (Ref: Man); ‘Income’ = Household Income; ‘Fin. W-b.’ = Financial Well-being; ‘Employment’ = Dichotomized Employment (Ref: Not Working); ‘Education’ = Education level; ‘Married’ = Dichotomized Marital Status (Ref: Not Married); ‘White’ = Dichotomized Ethnicity (Ref: Not White); ‘English’ = Dichotomized English as Home Language (Ref: No); ‘Children’ = Number of Children; ‘1st-time Parent’ = Dichotomized First-time Parent (Ref: No)

We next examined which sociodemographic factors predict parent preferences for psychologist and medical doctor coaches (see Fig. 4 for correlational relationships). Identifying as a woman, higher household income, lower perceived financial well-being, lower employment, identifying as White, primarily speaking English at home, and not being a first-time parent were all significantly associated with endorsing more program coaches. No sociodemographic characteristics were associated with endorsement of peer or community coaches. Binary logistic regression examining parent preferences for psychologist coaches was significant (χ^2^(8) = 49.879, *p* <.001). This model (Table 4a) showed that each increase in financial well-being was associated with less frequent endorsement of psychologist coaches (Exp(B) = 0.735, 95% CI [0.603, 0.895], *p* =.002). Those who identified as a woman (Exp(B) = 1.767, 95% CI [1.213, 2.573], *p* =.003) and White (Exp(B) = 1.920, 95% CI [1.346, 2.737], *p* <.001) also more frequently endorsed psychologist coaches. Binary logistic regression examining parent preferences for medical doctor coaches was significant (χ^2^(8) = 16.250, *p* =.039). This model (Table 4b) showed that each increase in household income was associated with more frequent endorsement of medical doctor coaches (Exp(B) = 1.167, 95% CI [1.057, 1.287], *p* =.002). Another model examining preferences for social worker coaches was significant overall, but no individual predictor was significant in the model (χ^2^(8) = 30.662, *p* <.001; Table 4c). Additional models examined preferences for peer and community coaches, but no model omnibus was significant, p’s > 0.05 (Tables 5d, 5e).

**Table 4:** Binary logistic regression model summary for sociodemographic predictors of program coach credential preferences (RQ 4)

Outcome	(a) Psychologist Coaches			(b) Medical Doctor Coaches			(c) Social Worker Coaches		
	Exp(B)	95% CI	*p*	Exp(B)	95% CI	*p*	Exp(B)	95% CI	*p*
Intercept	0.72		0.501	0.30		0.011	0.46		0.114
Gender Identity (ζ)	1.77	[1.21, 2.57]	0.003*^ζ^	1.22	[0.83, 1.79]	0.320	1.38	[0.93, 2.05]	0.113
Household Income	1.13	[1.03, 1.24]	0.011	1.17	[1.06, 1.29]	0.002*	1.14	[1.03, 1.25]	0.011
Financial Well-being	0.74	[0.60, 0.90]	0.002*	1.00	[0.83, 1.20]	0.993	0.75	[0.61, 0.93]	0.008
Employment (Ref: Not working)	-	-	-	-	-	-	-	-	-
Stay-at-home	0.52	[0.26, 1.04]	0.066	1.31	[0.66, 2.56]	0.439	0.41	[0.20, 0.85]	0.016
Working	0.85	[0.46, 1.56]	0.593	1.05	[0.57, 1.93]	0.871	0.93	[0.51, 1.70]	0.816
White	1.92	[1.35, 2.74]	< 0.001*	1.31	[0.93, 1.86]	0.124	1.26	[0.87, 1.82]	0.227
English as Home Language	1.27	[0.79, 2.05]	0.318	0.85	[0.54, 1.34]	0.486	1.63	[0.97, 2.73]	0.066
First-time Parent	1.18	[0.84, 1.67]	0.340	0.92	[0.66, 1.29]	0.637	0.74	[0.52, 1.05]	0.095

Note. ^*^ = *p*-value less than the Benjamini-Hochberg critical *p*-value for this effect. ^ζ^ = Effect significance differs from the supplementary model without multiple imputation. “Gender Identity” is a variable dichotomized ‘woman’ (1) or ‘man’ (0). “White,” “English as Home Language,” and “First-time Parent” are all variables dichotomized ‘Yes’ (1) or ‘No’ (0)

**Table 5:** Binary logistic regression model summary for sociodemographic predictors of program coach credential preferences (RQ 4)

Outcome	(d) Peer Coaches			(e) Community Coaches		
	Exp(B)	95% CI	*p*	Exp(B)	95% CI	*p*
Intercept	0.35		0.049	0.54		0.214
Gender Identity	0.92	[0.60, 1.41]	0.691	0.90	[0.59, 1.35]	0.594
Household Income	0.94	[0.84, 1.05]	0.280	0.95	[0.85, 1.06]	0.330
Financial Well-being	1.06	[0.86, 1.31]	0.611	1.01	[0.82, 1.24]	0.951
Employment (Ref: Not working)	-	-	-	-	-	-
Stay-at-home	1.16	[0.54, 2.47]	0.709	0.61	[0.30, 1.23]	0.169
Working	0.91	[0.46, 1.80]	0.786	0.62	[0.34, 1.15]	0.128
White	0.72	[0.48, 1.08]	0.113	1.13	[0.77, 1.65]	0.536
English as Home Language	1.30	[0.75, 2.27]	0.348	1.25	[0.72, 2.17]	0.424
First-time Parent	1.00	[0.68, 1.48]	0.983	1.24	[0.86, 1.80]	0.257

Note. ^*^ = *p*-value less than the Benjamini-Hochberg critical *p*-value for this effect. ^ζ^ = Effect significance differs from the supplementary model without multiple imputation. “Gender Identity” is a variable dichotomized ‘woman’ (1) or ‘man’ (0). “White,” “English as Home Language,” and “First-time Parent” are all variables dichotomized ‘Yes’ (1) or ‘No’ (0)

#### Which sociodemographic factors predict parent preferences for audio-visual content delivery?

**Fig. 5:**
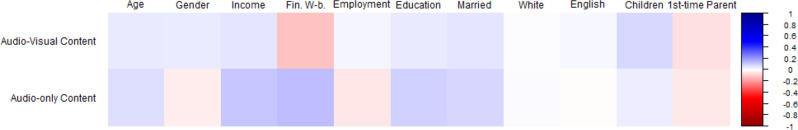
Bivariate correlations between sociodemographic factors and audio-visual content delivery preferences. Note. Square color represents correlation directionality (blue = positive, red = negative). Square color darkness represents correlation strength (darkest blue is *r* = 1.00, darkest red is *r* = -1.00, White is *r* =.00). Sociodemographic variables at top, Parent preferences at left. At top: ‘Age’ = Parent age in years; ‘Gender’ = Dichotomized Gender (Ref: Man); ‘Income’ = Household Income; ‘Fin. W-b.’ = Financial Well-being; ‘Employment’ = Dichotomized Employment (Ref: Not Working); ‘Education’ = Education level; ‘Married’ = Dichotomized Marital Status (Ref: Not Married); ‘White’ = Dichotomized Ethnicity (Ref: Not White); ‘English’ = Dichotomized English as Home Language (Ref: No); ‘Children’ = Number of Children; ‘1st-time Parent’ = Dichotomized First-time Parent (Ref: No)

We next examined which sociodemographic characteristics predict parent preferences for audio-visual and audio-only content delivery (see Fig. 5 for correlational relationships). Greater household income, higher education level, and being married were all significantly correlated with preferences for content delivery and were thus included in the model. Notably, lower perceived financial well-being was associated with preferences for audio-visual content delivery while higher perceived financial well-being was associated with preferences for audio-only content delivery. Binary logistic regression examining parent preferences for audio-visual content delivery was significant (χ^2^(4) = 16.928, *p* =.002). This model (Table 6a) found that each increase in financial well-being was associated with less frequent endorsement of audio-visual content delivery (Exp(B) = 0.720, 95% CI [0.602, 0.862], *p* <.001). The model examining audio-only content delivery had a significant omnibus but did not demonstrate any significant effects (χ^2^(4) = 15.462, *p* =.004; Table 6b).

**Table 6:** Binary logistic regression model summary for sociodemographic predictors of audio-visual content delivery preferences (RQ 5)

Outcome	(a) Audio-Visual Content			(b) Audio-only Content			
	Exp(B)	95% CI	*p*	Exp(B)	95% CI		*p*
Intercept	1.24		0.492	0.05			< 0.001
Household Income	1.10	[1.00, 1.21]	0.040	1.04		[0.93, 1.17]	0.485
Financial Well-being	0.72	[0.60, 0.86]	< 0.001*	1.31		[1.04, 1.65]	0.020
Education	1.06	[0.90, 1.25]	0.463	1.12		[0.90, 1.38]	0.311
Married	1.15	[0.75, 1.78]	0.516	1.46		[0.79, 2.71]	0.233

Note. ^*^ = *p*-value less than the Benjamini-Hochberg critical *p*-value for this effect. ^ζ^ = Effect significance differs from the supplementary model without multiple imputation. “Married” is a variable dichotomized ‘Yes’ (1) or ‘No’ (0)

#### Which sociodemographic factors predict number of barriers to program access?

**Fig. 6:**
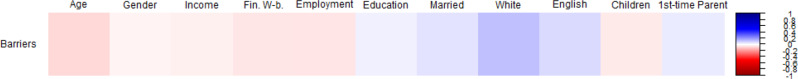
Bivariate correlations between sociodemographic factors and barriers to program access. Note. Square color represents correlation directionality (blue = positive, red = negative). Square color darkness represents correlation strength (darkest blue is *r* = 1.00, darkest red is *r* = -1.00, White is *r* =.00). Sociodemographic variables at top, Parent preferences at left. At top: ‘Age’ = Parent age in years; ‘Gender’ = Dichotomized Gender (Ref: Man); ‘Income’ = Household Income; ‘Fin. W-b.’ = Financial Well-being; ‘Employment’ = Dichotomized Employment (Ref: Not Working); ‘Education’ = Education level; ‘Married’ = Dichotomized Marital Status (Ref: Not Married); ‘White’ = Dichotomized Ethnicity (Ref: Not White); ‘English’ = Dichotomized English as Home Language (Ref: No); ‘Children’ = Number of Children; ‘1st-time Parent’ = Dichotomized First-time Parent (Ref: No)

Next, we examined which sociodemographic factors predict the number of reported program access barriers for parents (e.g., limited time, childcare, wi-fi, electronic devices; see Fig. 6 for correlational relationships). Younger parent age, identifying as a man, greater perceived financial well-being, lower employment, greater education level, and identifying as White were all significantly associated with reported barriers involving resources (e.g., time, childcare) and technology (e.g., wi-fi, device access). Negative binomial regression examining program access barriers was significant (χ^2^(11) = 28.460, *p* =.003). This model (Table 7) found that parents who identified as White reported more barriers than those who were not White (Exp(B) = 1.274, 95% CI [1.080, 1.504], *p* =.004). Interestingly, those with a graduate or professional degree (Exp(B) = 2.188, 95% CI [1.276, 3.755], *p* =.004), those with a Bachelor’s degree (Exp(B) = 2.100, 95% CI [1.249, 3.536], *p* =.005), and those who completed high school (Exp(B) = 2.181, 95% CI [1.285, 3.706], *p* =.004) reported more barriers than those who did not complete high school.

**Table 7:** Negative binomial regression model summary for sociodemographic predictors of barriers to program access (RQ 6)

Outcome	Barriers to Program Access		
	Exp(B)	95% CI	*p*
Intercept	1.55	[0.75, 3.21]	0.235
Age (ζ)	0.99	[0.98, 1.00]	.073^ζ^
Gender Identity	0.90	[0.76, 1.07]	0.230
Household Income	0.99	[0.95, 1.04]	0.693
Financial Well-being	0.93	[0.85, 1.01]	0.097
Employment (Ref: Not working)	-	-	-
Stay-at-home	0.77	[0.57, 1.05]	0.098
Working	0.92	[0.72, 1.19]	0.550
Employment (Ref: Working)	-	-	-
Stay-at-home	0.84	[0.66, 1.06]	0.144
Not working	1.08	[0.84, 1.40]	0.550
Education (Ref: Less than High School)	-	-	-
Graduate or Professional Degree (ζ)	2.19	[1.28, 3.76]	0.004*^ζ^
Bachelor’s Degree	2.10	[1.25, 3.54]	0.005*
College/Technical School	1.77	[1.05, 3.01]	0.034
High School	2.18	[1.29, 3.71]	0.004*
White	1.27	[1.08, 1.50]	0.004*

Note. ^*^ = *p*-value less than the Benjamini-Hochberg critical *p*-value for this effect. ^ζ^ = Effect significance differs from the supplementary model without multiple imputation. “Gender Identity” is a variable dichotomized ‘woman’ (1) or ‘man’ (0). “White” is a variable dichotomized ‘Yes’ (1) or ‘No’ (0)

#### Which sociodemographic factors predict parent preferences for peer shared identity?

**Fig. 7:**
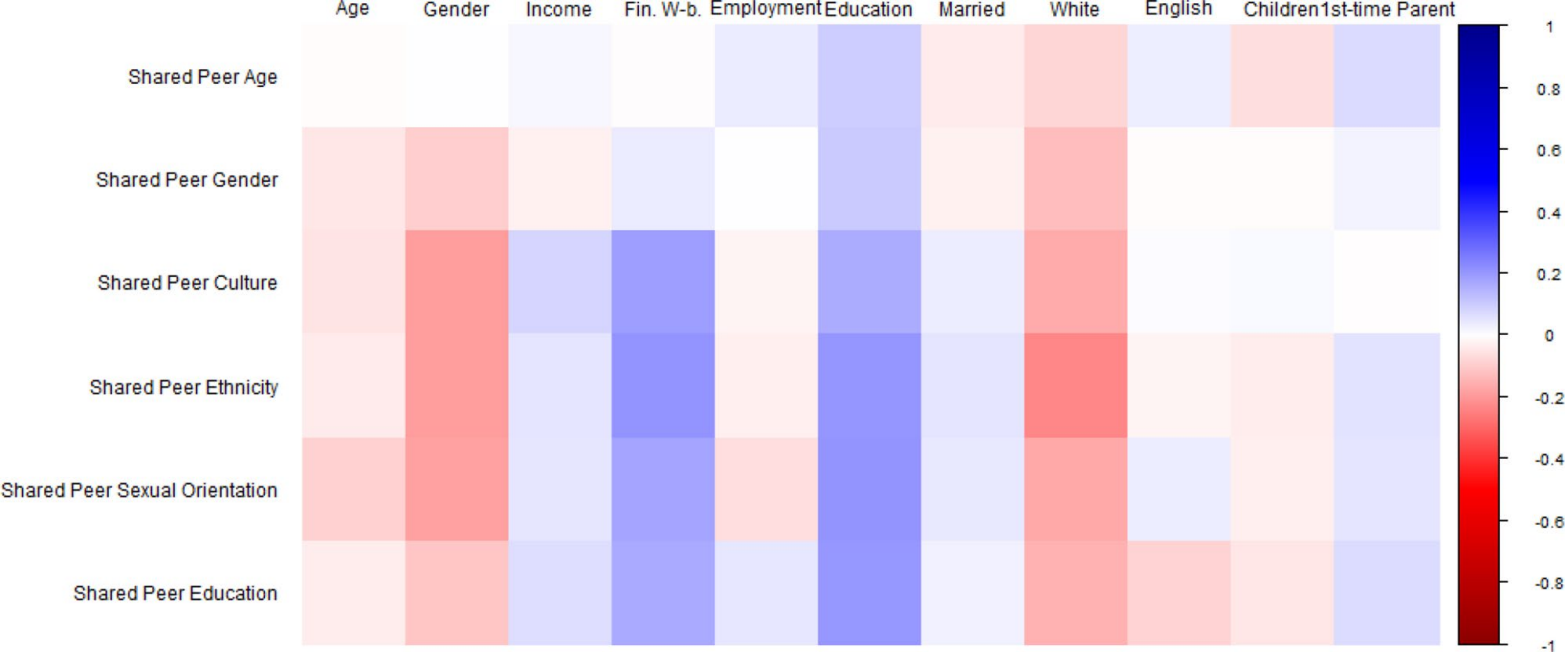
Bivariate correlations between sociodemographic factors and peer shared identity preferences. Note. Square color represents correlation directionality (blue = positive, red = negative). Square color darkness represents correlation strength (darkest blue is *r* = 1.00, darkest red is *r* = -1.00, White is *r* =.00). Sociodemographic variables at top, Parent preferences at left. At top: ‘Age’ = Parent age in years; ‘Gender’ = Dichotomized Gender (Ref: Man); ‘Income’ = Household Income; ‘Fin. W-b.’ = Financial Well-being; ‘Employment’ = Dichotomized Employment (Ref: Not Working); ‘Education’ = Education level; ‘Married’ = Dichotomized Marital Status (Ref: Not Married); ‘White’ = Dichotomized Ethnicity (Ref: Not White); ‘English’ = Dichotomized English as Home Language (Ref: No); ‘Children’ = Number of Children; ‘1st-time Parent’ = Dichotomized First-time Parent (Ref: No)

We next examined sociodemographic characteristics that predict parent preferences for peer shared identity (see Fig. 7 for correlational relationships). Younger parent age, identifying as a man, greater perceived financial well-being, greater education level, being married, being non-White, and being a first-time parent were all significantly correlated with greater preferences for program peers and coaches to share different aspects of their identities. Linear regression models examined sociodemographic predictors of preference for shared peer gender (F(8, 597) = 2.728, *p* =.006, R^2^ = 0.035; Table 8b), culture (F(8, 597) = 6.281, *p* <.001, R^2^ = 0.078; Table 8c), ethnicity (F(8, 597) = 9.455, *p* <.001, R^2^ = 0.112; Table 8d), sexual orientation (F(8, 597) = 7.537, *p* <.001, R^2^ = 0.092; Table 9e), and education level (F(8, 597) = 5.914, *p* <.001, R^2^ = 0.0873; Table 9f). The model examining preference for shared peer age was not significant (F(8, 597) = 1.753, *p* =.084, R^2^ = 0.023; Table 8a).

**Table 8:** Linear regression model summary for sociodemographic predictors of preferences for shared peer and coach identity (RQ 7, 8)

Outcome	(a) Shared Peer Age Identity				(b) Shared Peer Gender Identity				(c) Shared Peer Cultural Identity				(d) Shared Peer Ethnic Identity			
	B	β	t	*p*	B	β	t	*p*	B	β	t	*p*	B	β	t	*p*
Intercept	3.65		8.45	< 0.001	3.91		9.05	< 0.001	3.29		7.20	< 0.001	3.13		7.63	< 0.001
Age	0.00	− 0.02	-0.44	0.664	-0.02	− 0.08	-1.71	0.089	-0.01	− 0.05	-0.93	0.364	-0.01	− 0.04	-0.95	0.343
Gender Identity (ζ)	-0.03	− 0.01	-0.26	0.793	-0.30	− 0.11	-2.35	0.020	-0.43	− 0.16	-3.17	0.002*	-0.38	− 0.14	-2.40	.029^ζ^
Household Income	0.01	0.01	0.20	0.841	-0.02	− 0.03	-0.57	0.568	-0.02	− 0.02	-0.45	0.650	-0.05	− 0.08	-1.69	0.093
Financial Well-being (ζ)	-0.07	− 0.05	-1.11	0.269	-0.02	− 0.01	-0.27	0.790	0.12	0.09	1.62	0.113	0.15	0.11	2.38	.018^ζ^
Education (ζ)	0.13	0.10	1.79	0.085	0.18	0.14	2.76	0.007	0.17	0.14	2.51	.016^ζ^	0.19	0.16	2.90	0.006*
Married	-0.20	− 0.06	-1.39	0.165	-0.12	− 0.04	-0.64	0.530	-0.02	− 0.01	-0.16	0.877	0.00	0.00	0.02	0.983
White	-0.08	− 0.03	-0.55	0.589	-0.12	− 0.04	-0.93	0.354	-0.23	− 0.08	-1.78	0.079	-0.40	− 0.15	-3.53	< 0.001*
First-time Parent	0.08	0.03	0.75	0.452	-0.06	− 0.02	-0.47	0.640	-0.09	− 0.03	-0.67	0.506	0.00	0.00	0.03	0.974

Note. ^*^ = *p*-value less than the Benjamini-Hochberg critical *p*-value for this effect. ^ζ^ = Effect significance differs from the supplementary model without multiple imputation. “Gender Identity” is a variable dichotomized ‘woman’ (1) or ‘man’ (0). “Married,” “White,” and “First-time Parent” are all variables dichotomized ‘Yes’ (1) or ‘No’ (0)

**Table 9:** Linear regression model summary for sociodemographic predictors of preferences for shared peer and coach identity (RQ 7, 8)

Outcome	(e) Shared Peer Sexual Orientn.				(f) Shared Peer Education				(g) Shared Coach Age Identity				(h) Shared Coach Gender Identity			
	B	β	t	*p*	B	β	t	*p*	B	β	t	*p*	B	β	t	*p*
Intercept	3.57		7.86	< 0.001	3.09		6.25	< 0.001	3.29		7.71	< 0.001	3.32		8.09	< 0.001
Age	-0.02	− 0.10	-2.28	0.024	-0.01	− 0.06	-1.22	0.227	-0.01	− 0.07	-1.19	0.242	-0.02	− 0.10	-2.03	0.044
Gender Identity (ζ)	-0.49	− 0.16	-3.82	< 0.001*	-0.20	− 0.07	-1.55	0.121	-0.36	− 0.13	-2.67	.010^ζ^	-0.33	− 0.12	-2.17	0.040
Household Income	-0.05	− 0.07	-1.49	0.137	-0.01	− 0.01	-0.26	0.792	-0.04	− 0.05	-1.11	0.269	-0.02	− 0.03	-0.54	0.588
Financial Well-being	0.09	0.06	1.38	0.169	0.10	0.08	1.45	0.153	0.04	0.03	0.69	0.494	0.08	0.06	1.20	0.229
Education	0.25	0.19	3.44	0.002*	0.24	0.19	3.67	< 0.001*	0.21	0.18	3.43	< 0.001*	0.25	0.21	3.60	0.001*
Married	0.03	0.01	0.20	0.844	-0.04	− 0.01	-0.24	0.807	0.01	0.00	0.06	0.949	-0.06	− 0.02	-0.37	0.712
White	-0.23	− 0.08	-1.93	0.055	-0.17	− 0.06	-1.26	0.215	-0.02	− 0.01	-0.15	0.880	-0.05	− 0.02	-0.36	0.718
First-time Parent	-0.03	− 0.01	-0.25	0.799	0.00	0.00	0.01	0.989	0.16	0.06	1.32	0.190	0.01	0.00	0.05	0.960

Note. ^*^ = *p*-value less than the Benjamini-Hochberg critical *p*-value for this effect. ^ζ^ = Effect significance differs from the supplementary model without multiple imputation. “Gender Identity” is a variable dichotomized ‘woman’ (1) or ‘man’ (0). “Married,” “White,” and “First-time Parent” are all variables dichotomized ‘Yes’ (1) or ‘No’ (0)

Respondents who identified as a woman reported it being less important that their program peers share their cultural identity (β = − 0.153, t = -3.168, *p* =.002) and sexual orientation (β = − 0.166, t = -3.819, *p* <.001). Each increase in education level among respondents was associated with increased importance that their peers share their ethnic identity (β = 0.156, t = 2.903, *p* =.006), sexual orientation (β = 0.193, t = 3.435, *p* =.002) and education level (β = 0.191, t = 3.670, *p* <.001). Finally, respondents who identified as White reported decreased importance that their peers share their own ethnic identity (β = − 0.151, t = -3.530, *p* <.001).

#### Which sociodemographic factors predict parent preferences for coach shared identity?

**Fig. 8:**
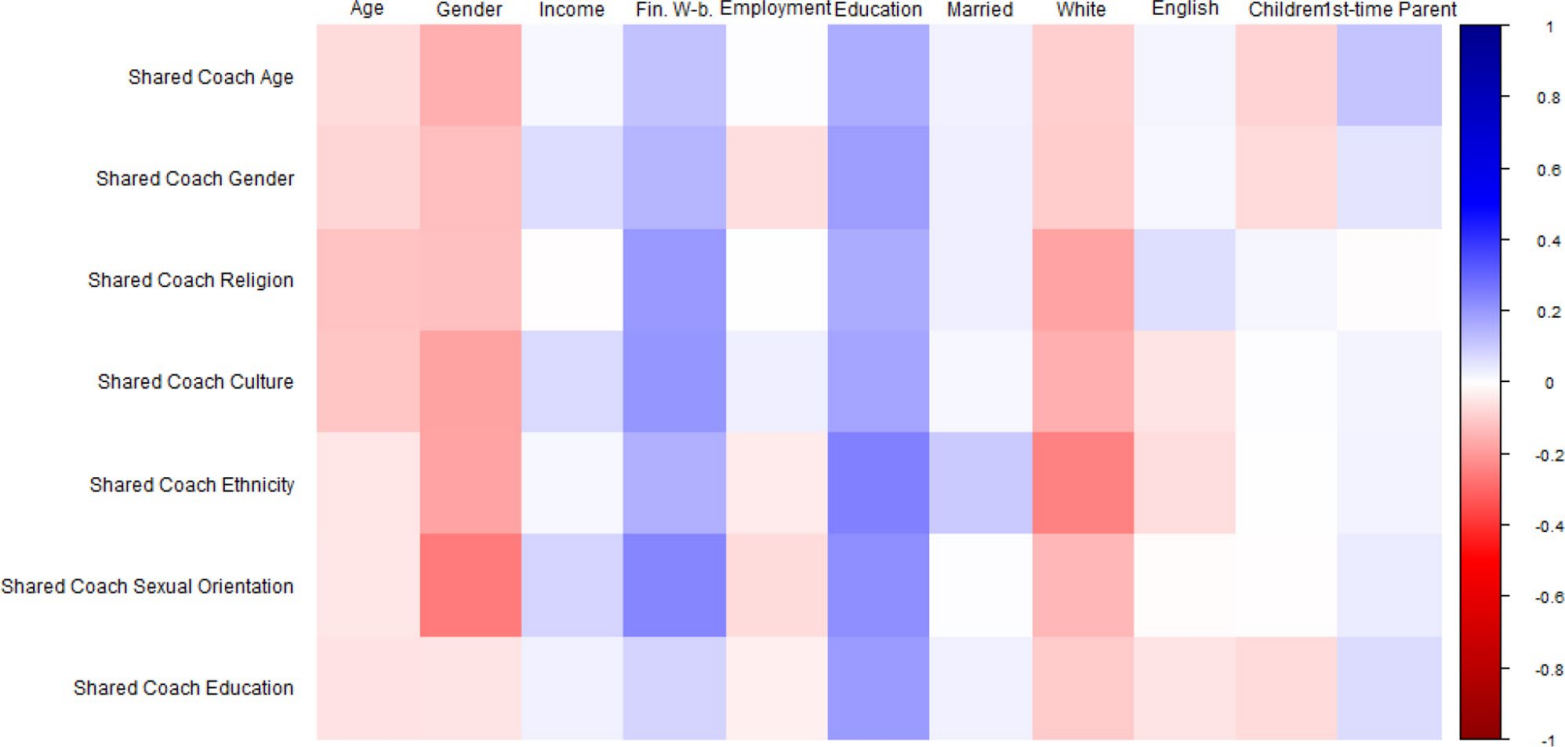
Bivariate correlations between sociodemographic factors and program coach shared identity preferences. Note. Square color represents correlation directionality (blue = positive, red = negative). Square color darkness represents correlation strength (darkest blue is *r* = 1.00, darkest red is *r* = -1.00, White is *r* =.00). Sociodemographic variables at top, Parent preferences at left. At top: ‘Age’ = Parent age in years; ‘Gender’ = Dichotomized Gender (Ref: Man); ‘Income’ = Household Income; ‘Fin. W-b.’ = Financial Well-being; ‘Employment’ = Dichotomized Employment (Ref: Not Working); ‘Education’ = Education level; ‘Married’ = Dichotomized Marital Status (Ref: Not Married); ‘White’ = Dichotomized Ethnicity (Ref: Not White); ‘English’ = Dichotomized English as Home Language (Ref: No); ‘Children’ = Number of Children; ‘1st-time Parent’ = Dichotomized First-time Parent (Ref: No)

We finally examined sociodemographic characteristics that predict parent preferences for program coach shared identity (see Fig. 8 for correlational relationships). Younger parent age, identifying as a man, greater perceived financial well-being, greater education level, being married, being non-White, and being a first-time parent were all significantly correlated with greater preferences for program peers and coaches to share different aspects of their identities. Linear regression models examined sociodemographic predictors of preference for shared coach age (F(8, 597) = 5.239, *p* <.001, R^2^ = 0.066; Table 9g), gender (F(8, 597) = 4.492, *p* <.001, R^2^ = 0.057; Table 9h), religion (F(8, 597) = 7.116, *p* <.001, R^2^ = 0.087; Table 10i), culture (F(8, 597) = 6.491, *p* <.001, R^2^ = 0.080; Table 10j), ethnicity (F(8, 597) = 10.655, *p* <.001, R^2^ = 0.125; Table 10k), sexual orientation (F(8, 597) = 10.981, *p* <.001, R^2^ = 0.128; Table 11l), and education level (F(8, 597) = 4.320, *p* <.001, R^2^ = 0.055; Table 11m).

**Table 10:** Linear regression model summary for sociodemographic predictors of preferences for shared peer and coach identity (RQ 7, 8)

Outcome	(i) Shared Coach Religion				(j) Shared Coach Cultural Identity				(k) Shared Coach Ethnic Identity			
	B	β	t	*p*	B	β	t	*p*	B	β	t	*p*
Intercept	3.48		7.10	< 0.001	3.64		9.08	< 0.001	3.37		6.93	< 0.001
Age	-0.02	− 0.12	-2.55	0.011	-0.02	− 0.11	-2.59	0.010	-0.01	− 0.07	-1.43	0.156
Gender Identity	-0.27	− 0.10	-2.09	0.039	-0.42	− 0.15	-3.46	< 0.001*	-0.40	− 0.14	-3.05	0.003*
Household Income (ζ)	-0.07	− 0.10	-1.89	0.064	-0.02	− 0.03	-0.50	0.621	-0.08	− 0.11	-2.10	.041^ζ^
Financial Well-being (ζ)	0.17	0.13	2.63	.009^ζ^	0.13	0.10	2.09	0.037	0.07	0.05	0.78	0.449
Education	0.21	0.17	3.45	< 0.001*	0.20	0.16	3.45	< 0.001*	0.26	0.21	3.92	< 0.001*
Married	-0.08	− 0.02	-0.44	0.660	-0.08	− 0.02	-0.50	0.620	0.17	0.05	1.12	0.263
White (ζ)	-0.24	− 0.09	-1.95	.053^ζ^	-0.21	− 0.08	-1.62	0.112	-0.43	− 0.16	-3.38	0.001*
First-time Parent	-0.18	− 0.07	-1.30	0.203	-0.12	− 0.04	-0.87	0.393	-0.08	− 0.03	-0.73	0.467

Note. ^*^ = *p*-value less than the Benjamini-Hochberg critical *p*-value for this effect. ^ζ^ = Effect significance differs from the supplementary model without multiple imputation. “Gender Identity” is a variable dichotomized ‘woman’ (1) or ‘man’ (0). “Married,” “White,” and “First-time Parent” are all variables dichotomized ‘Yes’ (1) or ‘No’ (0)

**Table 11:** Linear regression model summary for sociodemographic predictors of preferences for shared peer and coach identity (RQ 7, 8)

Outcome	(l) Shared Coach Sexual Orientn.				(m) Shared Coach Education			
	B	β	t	*p*	B	β	t	*p*
Intercept	3.20		6.95	< 0.001	3.40		7.77	< 0.001
Age	-0.01	− 0.07	-1.51	0.133	-0.02	− 0.08	-1.69	0.093
Gender Identity	-0.58	− 0.20	-4.15	< 0.001*	-0.15	− 0.05	-1.29	0.197
Household Income	-0.03	− 0.04	-0.87	0.383	-0.02	− 0.02	-0.43	0.669
Financial Well-being (ζ)	0.15	0.11	1.97	.058^ζ^	0.04	0.03	0.59	0.558
Education	0.26	0.21	4.44	< 0.001*	0.25	0.20	3.74	< 0.001*
Married	-0.24	− 0.07	-1.34	0.189	0.08	0.02	0.50	0.622
White	-0.16	− 0.05	-1.33	0.185	-0.04	− 0.01	-0.35	0.724
First-time Parent	-0.07	− 0.02	-0.56	0.579	0.06	0.02	0.40	0.691

Note. ^*^ = *p*-value less than the Benjamini-Hochberg critical *p*-value for this effect. ^ζ^ = Effect significance differs from the supplementary model without multiple imputation. “Gender Identity” is a variable dichotomized ‘woman’ (1) or ‘man’ (0). “Married,” “White,” and “First-time Parent” are all variables dichotomized ‘Yes’ (1) or ‘No’ (0)

Respondents who identified as a woman reported it being less important that their program coaches share their cultural identity (β = − 0.150, t = -3.457, *p* <.001), ethnic identity (β = − 0.142, t = -3.049, *p* =.003), and sexual orientation (β = − 0.197, t = -4.149, *p* <.001). Each increase in education was associated with increased importance that coaches share their age (β = 0.176, t = 3.432, *p* <.001), gender (β = 0.203, t = 3.595, *p* =.001), religious identity (β = 0.164, t = 3.446, *p* <.001), cultural identity (β = 0.159, t = 3.446, *p* <.001), ethnic identity (β = 0.208, t = 3.921, *p* <.001), sexual orientation (β = 0.205, t = 4.437, *p* <.001), and education level (β = 0.208, t = 3.743, *p* <.001). Finally, respondents who identified as White reported it being less important that their program coaches share their ethnic identity (β = − 0.159, t = -3.377, *p* =.001).

## Discussion

This novel research examined what the general Canadian population of parents may want in parent mental health programs. Parents overall desired eHealth programs that can be accessed via the web, that are 2–4 weeks in duration, led by a psychologist, and that have both audio and visual components. The overall preference for an “expert” leading a program (e.g., psychologist, medical doctor, social worker), as opposed to a community member or peer coach, speaks to how the average parent may trust those who have training, education, or expertise in parenting and mental health to run these programs. Additionally, psychologists were the only choice that was endorsed by a (slight) majority of respondents. Medical doctor was the second-most preferred but was only endorsed by 40.5%. This might be because we framed the hypothetical program as a “family mental health” program, and thus participants chose psychologist, but further research examining why parents prefer certain experts over others would be needed to make sure. It was additionally found that no sociodemographic characteristics were associated with endorsement of peer or community coaches. This contrasts with some research which has demonstrated that peer-led parenting supports have been effective in improving factors like parenting skills and reducing variables like parenting stress, as well as being rated as satisfactory by participants [65, 66]. It is worth noting that these were parent support groups, which were not necessarily mental health programming but had strong themes of parent mental health. Regardless, this is informative as a foundation for a program that could be desirable to most Canadian parents, but it is also important to consider individual differences in parent preferences so that programs can be designed for the maximal benefit of all of parents.

Program features were not always preferred by a clear majority of parents. For example, there were mixed preferences regarding whether parents wanted a self-directed or peer-driven program, a synchronous or asynchronous program, or one with open or tailored content. There were also mixed preferences for how important it was that parents had aspects of their identity in common with other peers and program coaches. It might be that parents generally do not have a clear preference for one feature or another. It could also be that there are other factors at play in terms of shared identities that we did not capture or measure. One program feature that was notably preferred by certain parents was content delivery. Parents who reported lower perceived financial well-being also preferred audio-visual content while those with higher perceived financial well-being preferred audio-only content. This could be for many reasons, speaking to the different ways that parents might want to engage with content. For all of these program features, future research investigating why specific parents have these preferences would be needed to confirm the reasons for them.

In asking parents about barriers that prevent them from accessing a parent mental health program, the two most endorsed were lack of time and wi-fi capabilities, such as slow internet. Arranging childcare was also noted as a significant barrier by more than one in five participants. Thus, practical barriers regarding how one might access a parent mental health program seem to be a prevalent concern. This is aligned with literature citing lower income, lack of time, and lack of access to internet as barriers to starting and maintaining participation in parent mental health programs [21, 24, 25, 30, 34–36].

It is important that researchers and program developers consider ways to address accessibility concerns (e.g., reducing financial cost, addressing ways to circumvent a lack of internet access, considering asynchronous programs for parents with busy schedules) to lessen barriers and maximize parent participation. For example, some research found parents with lower income prefer the use of social media or parenting apps to access parenting information [18, 19], which may speak to a desire to access this content in a self-directed manner and have access to content for as long as possible, unattached to attending a “class” or “program”. Also possible is that these parents view social media and apps as free or low cost and classes or programs as more expensive. To meet the needs of Canada’s diverse population, it may also be helpful to address accessibility by translating materials into various languages or consult parents from various cultures in developing materials. Addressing these notable barriers that Canadian parents must face is a critical first step in making programs safer and more beneficial to families.

Based on this survey, participants with certain sociodemographic characteristics also had differential preferences for program features and were more likely to report certain barriers. The most robust sociodemographic characteristics associated with program preferences were parent gender, household income, perception of financial well-being, education, and ethnicity. These findings may inform more inclusive and accessible program development for parents who are facing specific barriers to accessing resources. For example, parents who identified as White preferred longer parent mental health programs. This converges with some previous literature suggesting that American parents who identified as White had greater preference for longer family-centred programming than did parents who identified as non-White [67]. Future qualitative research should examine why this might be, though it is possible that parents who identify as White perceive more resources, fewer occupational time constraints, and lower parenting stress, thus making longer programming more feasible [68, 69]. Longer programs may provide greater opportunity for knowledge sharing and uptake, which may lead to greater and longer lasting benefits for parents. Thus, future research should identify specific barriers preventing some parents from being able to fully participate in longer or more extensive programs.

The two most notable sociodemographic characteristics that were significantly correlated with program access barriers were parent education and identifying as White. Contrary to expectations, parents who reported higher levels of education and who identified as White reported more barriers to program access. This is interesting, as these parents also reported higher household income, better perceived financial well-being, and were more frequently married, suggesting they may have fewer challenges accessing programs. However, it is possible that parents with more resources may have greater opportunity to seek out resources, which may heighten their awareness of just how limited general mental health and parent mental health programs are. Further, parents with greater resources may be looking for different types of programs (e.g., individual therapy) than those with fewer resources and may genuinely encounter greater barriers to accessing these services (e.g., waitlists). Further research is necessary to examine this hypothesis.

One great strength and a novel aspect of this study was the timing of data collection in 2023. The Canadian population was more into the recovery phase of the COVID-19 pandemic, meaning that the present study’s findings might be longer lasting than what parents may have wanted during the pandemic (e.g., short-term, urgent needs that may have been more present during quarantine protocols as opposed to afterward). Additionally, the sample was relatively diverse (although not fully representative of Canada’s population). Respondents were situated across all Canadian provinces as well as the Yukon territory and identified across a range of ethnic identities. There was also variability in responses of financial well-being (some participants reporting being at floor, others at ceiling) and household income. The modal participant reporting household income at $70,000-$100,000, which would be at or below the $100,000 median household income in Ontario, where most participants in this sample reside [70]. Additionally, many reported being at each end of the household income distribution, with 16.60% reporting a household income below $40,000 and 14.60% reporting a household income above $150,000.

However, some limitations exist that need to be considered when interpreting these results. This study was completed using a self-report survey, meaning that participant answers were limited to the options presented to them. The survey was also distributed via the internet, meaning that families who are not regularly using or able to access the internet were likely excluded from our sample. In the future, research recruitment methods should include approaches that allow this subsample of parents to be involved (e.g., in-person recruitment at community centres, with paper copies or devices provided to parents to complete the survey). Additionally, the majority of participants identified as White, women, married or common-law, having full-time employment, having obtained at least one post-secondary degree, and the average participant also listed an average degree of financial well-being (although there was a lot of variability in responses). This speaks to how the sample could be diverse but not fully representative of Canada’s population. Additionally, we did not explicitly define what a parent mental health program was to participants in the survey. The accuracy and consistency of participant responses may have been better if we had defined parent mental health programs more specifically.

To enhance the external validity of future research, similar work should employ more diverse and representative sampling methods, such as random sampling, and targeted recruitment approaches to obtain information from underrepresented families to ensure a more accurate reflection of the broader population of parents with young children. Future research could also include semi-structured qualitative interviews to obtain more detailed information about the program preferences of parents to young children. It is possible that unconsidered program preferences and richer stories of parent experiences may emerge from such a qualitative or mixed methods design. Additionally, understanding why parents have certain preferences may inform how to adapt and create future programming.

## Conclusion

The purpose of this exploratory research was to investigate what specific factors and items are preferred by Canadian parents for online parent mental health programs. The study benefits from a large and diverse sample. Many preferences and barriers were reported amongst respondents, thus highlighting the critical need to create programs that are more accessible and inclusive for parents so that they can be the most effective for diverse families based on these differential preferences. The preferences found within this research (e.g., a preference for longer versus shorter parent mental health programs) may speak to a need to address barriers such as time constraints or financial strain for parents. Thus, this study is a novel and important step towards creating more accessible telehealth services and following guidelines, such as SPOR, by ensuring that we engage parents who need these services in the research aiming to support them.

## Electronic supplementary material

Below is the link to the electronic supplementary material.

Supplementary Material 1

## Data Availability

De-identified individual-level self-report data and SPSS syntax that underlie manuscript findings will be made available upon reasonable request. Requests for data should be directed jointly to the study co-senior authors (TH and LER) at tasmia.hai@mcgill.ca and leslie.roos@umanitoba.ca. All requests should detail the reason for the request and describe how the data will be used.

## Abbreviations

ATA: American Telemedicine Association
APA: American Psychiatric Association
CI: Condition Index
CIHR: Canadian Institute of Health Research
REDCap: Research Electronic Data Capture
SPOR: Strategies for Patient-Oriented Research
SPSS: Statistical Package for Social Science
VIF: Variance Inflation Factor
