# Supplementary material for: Parent and caregiver preferences for eHealth programs
Source: BMC Public Health. 2025 Jul 3;25:2307. doi: 10.1186/s12889-025-22612-8 (PMC12225051; doi:10.1186/s12889-025-22612-8)
Supplement: Supplementary file 1 — Supplementary Material 1 [file 12889_2025_22612_MOESM1_ESM.docx]

**Supplementary Materials**

**Parent Sociodemographics and eHealth Program Preference Descriptives**

*Sociodemographic Characteristics of the Sample*

| Variable | *n* | *M* | *SD* | Min | Max |
| --- | --- | --- | --- | --- | --- |
| Respondent Age | 603 | 33.88 | 6.87 | 18.67 | 64.33 |
| Gender Identity | - | - | - | - | - |
| Man | 217 | 35.8% |  |  |  |
| Woman | 384 | 63.4% |  |  |  |
| Non-binary | 1 | 0.2% |  |  |  |
| Two-spirit | 3 | 0.5% |  |  |  |
| Household Income | 583 | 4.27 | 1.99 | 1 | 9 |
| $0 to $19,999 (1) | 33 | 5.4% |  |  |  |
| $20,000 to $39,999 (2) | 68 | 11.2% |  |  |  |
| $40,000 to $69,999 (3) | 112 | 18.5% |  |  |  |
| $70,000 to $99,999 (4) | 166 | 27.4% |  |  |  |
| $100,000 to $124,999 (5) | 71 | 11.7% |  |  |  |
| $125,000 to $149,999 (6) | 45 | 7.4% |  |  |  |
| $150,000 to $174,999 (7) | 32 | 5.3% |  |  |  |
| $175,000+ (8, 9) | 56 | 9.3% |  |  |  |
| Financial Well-being | 601 | 3.00 | 1.01 | 1 | 5 |
| Employment | - | - | - | - | - |
| Working full-time | 375 | 61.9% |  |  |  |
| Working part-time | 74 | 12.2% |  |  |  |
| Unemployed | 28 | 4.6% |  |  |  |
| On leave | 22 | 3.6% |  |  |  |
| Retired | 3 | 0.5% |  |  |  |
| Full-time or Part-time student | 16 | 2.7% |  |  |  |
| Homemaker or stay-at-home parent | 122 | 20.1% |  |  |  |
| Education | 600 | - | - | - | - |
| Some high school, no diploma | 21 | 3.5% |  |  |  |
| High school diploma | 117 | 19.3% |  |  |  |
| Diploma from trade/technical/vocational training | 47 | 7.8% |  |  |  |
| Diploma from a CEGEP | 43 | 7.1% |  |  |  |
| Associate degree or undergraduate certificate | 56 | 9.2% |  |  |  |
| Bachelors degree | 204 | 33.7% |  |  |  |
| Master’s degree | 84 | 13.9% |  |  |  |
| Doctoral or Professional degree (e.g., PhD, EdD, MD, JD) | 28 | 4.7% |  |  |  |
| Marital Status | - | - | - | - | - |
| Married or Common-law relationship | 480 | 79.2% |  |  |  |
| Single, never married | 98 | 16.2% |  |  |  |
| Widowed, Divorced, or Separated | 26 | 4.3% |  |  |  |
| White | 603 | 54.9% |  |  |  |
| Ethnicity | 606 | - | - | - | - |
| White Canadian or White American | 316 | 52.1% |  |  |  |
| South Asian | 70 | 11.6% |  |  |  |
| White European | 62 | 10.2% |  |  |  |
| East Asian | 48 | 7.9% |  |  |  |
| Indigenous (e.g., First Nations, Métis, Inuk) | 37 | 6.1% |  |  |  |
| Latin American | 26 | 4.3% |  |  |  |
| Black African | 23 | 3.8% |  |  |  |
| Southeast Asian | 22 | 3.6% |  |  |  |
| Black Caribbean | 18 | 3.0% |  |  |  |
| Middle Eastern | 15 | 2.5% |  |  |  |
| Black Canadian or African-American | 14 | 2.3% |  |  |  |
| Indo-Caribbean | 6 | 1.0% |  |  |  |
| Other | 10 | 1.7% |  |  |  |
| Prefer not to Answer | 3 | 0.5% |  |  |  |
| Speaks English Primarily at Home | 606 | 83.8% |  |  |  |
| Languages Spoken at Home | 606 | - | - | - | - |
| English | 598 | 98.7% |  |  |  |
| French | 107 | 17.7% |  |  |  |
| Spanish | 28 | 4.6% |  |  |  |
| Punjabi | 22 | 3.6% |  |  |  |
| Cantonese | 21 | 3.5% |  |  |  |
| Mandarin | 18 | 3.0% |  |  |  |
| Iranian Persian | 13 | 2.1% |  |  |  |
| Urdu | 13 | 2.1% |  |  |  |
| Tagalog | 12 | 2.0% |  |  |  |
| Arabic | 11 | 1.8% |  |  |  |
| Russian | 5 | 0.8% |  |  |  |
| Indigenous language (e.g., Cree, Ojibway) | 5 | 0.8% |  |  |  |
| Other | 40 | 6.6% |  |  |  |
| Number of Children | 604 | 1.73 | 0.91 | 1 | 6 |

*Note.* Statistics in this table describe parent responses prior to multiple imputation. Ethnicity and language total >100% because participants were free to select all applicable responses. A slight majority of participants identified as being only white (54.9%; *n* = 331). Most participants identified as speaking English Primarily at Home (83.8%; *n* = 508).

*Bivariate Correlations*

|  | 1. | 2. | 3. | 4. | 5. | 6. | 7. | 8. | 9. | 10. | 11. | 12. | 13. | 14. | 15. | 16. | 17. | 18. | 19. | 20. | 21. | 22. | 23. | 24. | 25. | 26. | 27. | 28. | 29. | 30. | 31. | 32. | 33. | 34. | 35. | 36. | 37. | 38. | 39. | 40. |
| --- | --- | --- | --- | --- | --- | --- | --- | --- | --- | --- | --- | --- | --- | --- | --- | --- | --- | --- | --- | --- | --- | --- | --- | --- | --- | --- | --- | --- | --- | --- | --- | --- | --- | --- | --- | --- | --- | --- | --- | --- |
| 2. | -.08^†^ |  |  |  |  |  |  |  |  |  |  |  |  |  |  |  |  |  |  |  |  |  |  |  |  |  |  |  |  |  |  |  |  |  |  |  |  |  |  |  |
| 3. | .13^*^ | -.17^**^ |  |  |  |  |  |  |  |  |  |  |  |  |  |  |  |  |  |  |  |  |  |  |  |  |  |  |  |  |  |  |  |  |  |  |  |  |  |  |
| 4. | -.13^*^ | -.28^**^ | .37^**^ |  |  |  |  |  |  |  |  |  |  |  |  |  |  |  |  |  |  |  |  |  |  |  |  |  |  |  |  |  |  |  |  |  |  |  |  |  |
| 5. | -.08 | .12^‡^ | -.10^†^ | -.05 |  |  |  |  |  |  |  |  |  |  |  |  |  |  |  |  |  |  |  |  |  |  |  |  |  |  |  |  |  |  |  |  |  |  |  |  |
| 6. | .16^**^ | -.02 | .35^**^ | .22^**^ | -.15^**^ |  |  |  |  |  |  |  |  |  |  |  |  |  |  |  |  |  |  |  |  |  |  |  |  |  |  |  |  |  |  |  |  |  |  |  |
| 7. | .05 | -.06 | .27^**^ | .12^*^ | -.01 | .28^**^ |  |  |  |  |  |  |  |  |  |  |  |  |  |  |  |  |  |  |  |  |  |  |  |  |  |  |  |  |  |  |  |  |  |  |
| 8. | .06 | .03 | .01 | -.09^†^ | .08 | -.21^**^ | -.00 |  |  |  |  |  |  |  |  |  |  |  |  |  |  |  |  |  |  |  |  |  |  |  |  |  |  |  |  |  |  |  |  |  |
| 9. | -.01 | -.04 | -.02 | -.03 | -.04 | -.16^**^ | -.06 | .20^**^ |  |  |  |  |  |  |  |  |  |  |  |  |  |  |  |  |  |  |  |  |  |  |  |  |  |  |  |  |  |  |  |  |
| 10. | .17^**^ | .04 | -.05 | -.05 | .06 | -.18^**^ | .04 | .10^†^ | .04 |  |  |  |  |  |  |  |  |  |  |  |  |  |  |  |  |  |  |  |  |  |  |  |  |  |  |  |  |  |  |  |
| 11. | -.19^**^ | -.03 | -.04 | .03 | -.04 | .11^‡^ | -.10^†^ | -.10^†^ | -.01 | -.80^**^ |  |  |  |  |  |  |  |  |  |  |  |  |  |  |  |  |  |  |  |  |  |  |  |  |  |  |  |  |  |  |
| 12. | .00 | .14^**^ | -.07 | -.20^**^ | -.03 | .01 | -.02 | .12^‡^ | .03 | .03 | -.03 |  |  |  |  |  |  |  |  |  |  |  |  |  |  |  |  |  |  |  |  |  |  |  |  |  |  |  |  |  |
| 13. | .08 | -.04 | .13* | -.03 | -.04 | .08^†^ | .12^*^ | .04 | .06 | .00 | -.00 | .33^**^ |  |  |  |  |  |  |  |  |  |  |  |  |  |  |  |  |  |  |  |  |  |  |  |  |  |  |  |  |
| 14. | .05 | .09^†^ | -.04 | -.13^*^ | -.07 | .01 | -.02 | .11^†^ | .00 | -.02 | -.03 | .50^**^ | .26^**^ |  |  |  |  |  |  |  |  |  |  |  |  |  |  |  |  |  |  |  |  |  |  |  |  |  |  |  |
| 15. | -.04 | .07 | .01 | -.05 | -.01 | -.02 | .08^†^ | .04 | .07 | .02 | -.03 | .42^**^ | .43^**^ | .29^**^ |  |  |  |  |  |  |  |  |  |  |  |  |  |  |  |  |  |  |  |  |  |  |  |  |  |  |
| 16. | -.03 | .09 | .02 | -.13^‡^ | -.03 | -.07 | -.13^*^ | .18^**^ | .06 | .09^†^ | -.05 | .22^**^ | .10^†^ | .22^**^ | .10^†^ |  |  |  |  |  |  |  |  |  |  |  |  |  |  |  |  |  |  |  |  |  |  |  |  |  |
| 17. | -.09^†^ | -.12^‡^ | -.14^*^ | .08 | .03 | .02 | -.04 | -.06 | -.08 | .01 | .04 | -.12^‡^ | -.13^*^ | -.13^*^ | -.12^‡^ | -.07 |  |  |  |  |  |  |  |  |  |  |  |  |  |  |  |  |  |  |  |  |  |  |  |  |
| 18. | .02 | -.07 | .07 | .11^†^ | -.09^†^ | .00 | .03 | .02 | -.04 | .01 | -.02 | -.00 | .01 | .05 | -.02 | .04 | .11^†^ |  |  |  |  |  |  |  |  |  |  |  |  |  |  |  |  |  |  |  |  |  |  |  |
| 19. | -.01 | -.01 | .03 | .04 | -.04 | .06 | .06 | .03 | .07 | .07 | -.04 | -.03 | .00 | -.02 | .00 | -.02 | .20^**^ | -.07 |  |  |  |  |  |  |  |  |  |  |  |  |  |  |  |  |  |  |  |  |  |  |
| 20. | -.04 | -.05 | .04 | .04 | .01 | .01 | .04 | .01 | -.07 | .06 | -.04 | -.09 | -.09 | -.16^**^ | -.01 | -.08 | .11^†^ | -.06 | -.02 |  |  |  |  |  |  |  |  |  |  |  |  |  |  |  |  |  |  |  |  |  |
| 21. | -.04 | .13^*^ | .05 | -.14^**^ | -.06 | .01 | .03 | .17^**^ | .07 | -.06 | .02 | .36^**^ | .34^**^ | .31^**^ | .27^**^ | .13^‡^ | -.23^**^ | -.01 | .07 | -.13^*^ |  |  |  |  |  |  |  |  |  |  |  |  |  |  |  |  |  |  |  |  |
| 22. | .00 | .04 | .13^*^ | .03 | .04 | .06 | .01 | .07 | -.02 | .04 | -.04 | .25^**^ | .24^**^ | .15^**^ | .26^**^ | .09^†^ | -.13^*^ | .03 | -.02 | -.04 | .24^**^ |  |  |  |  |  |  |  |  |  |  |  |  |  |  |  |  |  |  |  |
| 23. | .04 | .07 | .08^†^ | -.08 | -.10^†^ | -.01 | -.02 | .08 | .09^†^ | .07 | -.08 | .36^**^ | .29^**^ | .26^**^ | .26^**^ | .15^*^ | -.14^**^ | .04 | -.10^†^ | -.10^†^ | .28^**^ | .21^**^ |  |  |  |  |  |  |  |  |  |  |  |  |  |  |  |  |  |  |
| 24. | -.05 | -.01 | -.05 | .01 | .02 | -.00 | -.07 | -.06 | .03 | -.02 | .01 | .17^**^ | .10^†^ | .09^†^ | .18^**^ | .10^†^ | .17^**^ | -.05 | -.07 | -.04 | .03 | -.01 | .07 |  |  |  |  |  |  |  |  |  |  |  |  |  |  |  |  |  |
| 25. | -.02 | -.00 | -.06 | -.01 | -.05 | -.03 | -.02 | .02 | .04 | -.06 | .05 | .31^**^ | .27^**^ | .17^**^ | .20^**^ | .04 | -.03 | .03 | .00 | -.06 | .19^**^ | .03 | .22^**^ | .23^**^ |  |  |  |  |  |  |  |  |  |  |  |  |  |  |  |  |
| 26. | .04 | .04 | .05 | -.12^*^ | .02 | .04 | .05 | .01 | .02 | .07 | -.06 | .29^**^ | .25^**^ | .19^**^ | .17^**^ | .07 | -.14^*^ | -.05 | -.03 | .02 | .23^**^ | .18^**^ | .22^**^ | .10^†^ | .15^**^ |  |  |  |  |  |  |  |  |  |  |  |  |  |  |  |
| 27. | .06 | -.04 | .11^‡^ | .13^*^ | -.05 | .09^†^ | .08 | .01 | -.01 | .03 | -.04 | .13^*^ | .14^**^ | .14^**^ | .16^**^ | .08 | -.07 | .05 | .04 | -.03 | .07 | .11^†^ | .07 | .07 | .11^‡^ | -.07 |  |  |  |  |  |  |  |  |  |  |  |  |  |  |
| 28. | -.07 | -.02 | -.03 | -.05 | -.05 | .03 | .05 | .12^*^ | .07 | -.04 | .04 | .32^**^ | .28^**^ | .23^**^ | .28^**^ | .04 | .02 | .07 | .10^†^ | -.10^†^ | .30^**^ | .18^**^ | .22^**^ | .11^‡^ | .23^**^ | .18^**^ | .18^**^ |  |  |  |  |  |  |  |  |  |  |  |  |  |
| 29. | -.01 | .00 | .02 | -.00 | .04 | .10^†^ | -.04 | -.08 | .03 | -.06 | .07 | .06 | .04 | .02 | .06 | .05 | .09^†^ | .04 | -.09^†^ | .06 | -.06 | -.04 | -.06 | .11^‡^ | .09^†^ | .00 | .04 | -.01 |  |  |  |  |  |  |  |  |  |  |  |  |
| 30. | -.05 | -.10^†^ | -.03 | .04 | .00 | .10^†^ | -.03 | -.13^*^ | -.01 | -.01 | .02 | -.04 | .03 | -.00 | .02 | -.03 | .17^**^ | .07 | -.06 | .07 | -.08 | -.06 | -.07 | .08 | .02 | -.03 | .01 | -.00 | .58^**^ |  |  |  |  |  |  |  |  |  |  |  |
| 31. | -.05 | -.19^**^ | .08 | .19^**^ | -.02 | .16^**^ | .03 | -.16^**^ | .01 | .01 | -.00 | -.12^*^ | .02 | -.11^‡^ | .00 | -.00 | .22^**^ | .14^*^ | -.01 | .08 | -.15^**^ | -.06 | -.08 | -.01 | -.05 | -.09^†^ | -.02 | -.04 | .37^**^ | .57^**^ |  |  |  |  |  |  |  |  |  |  |
| 32. | -.04 | -.19^**^ | .05 | .21^**^ | -.03 | .21^**^ | .05 | -.23^**^ | -.02 | -.04 | .06 | -.12^*^ | -.00 | -.10^†^ | -.01 | -.06 | .23^**^ | .10^†^ | .03 | .08 | -.19^**^ | -.09^†^ | -.13^*^ | -.02 | -.02 | -.16^**^ | .06 | -.05 | .34^**^ | .52^**^ | .71^**^ |  |  |  |  |  |  |  |  |  |
| 33. | -.09^†^ | -.18^**^ | .05 | .18^**^ | -.06 | .21^**^ | .04 | -.17^**^ | .04 | -.03 | .05 | -.11^‡^ | -.05 | -.12^‡^ | -.01 | -.06 | .22^**^ | .07 | .04 | .09^†^ | -.16^**^ | -.11^†^ | -.13^*^ | -.01 | -.04 | -.10^†^ | .06 | -.01 | .30^**^ | .51^**^ | .59^**^ | .70^**^ |  |  |  |  |  |  |  |  |
| 34. | -.04 | -.11^†^ | .06 | .17^**^ | .05 | .20^**^ | .03 | -.15^**^ | -.09 | -.05 | .07 | -.15^**^ | -.07 | -.14^**^ | -.07 | -.03 | .25^**^ | .10^†^ | -.00 | .05 | -.17^**^ | -.07 | -.12^‡^ | .01 | -.07 | -.08 | .05 | -.08 | .29^**^ | .40^**^ | .49^**^ | .52^**^ | .49^**^ |  |  |  |  |  |  |  |
| 35. | -.07 | -.16^**^ | .01 | .12^‡^ | .00 | .16^**^ | .03 | -.09^†^ | .02 | -.09 | .11^‡^ | -.10^†^ | .01 | -.09^†^ | -.03 | .06 | .25^**^ | .10^†^ | .08 | .07 | -.18^**^ | -.11^†^ | -.10^†^ | .03 | -.03 | -.08 | .08 | -.02 | .43^**^ | .53^**^ | .48^**^ | .51^**^ | .49^**^ | .55^**^ |  |  |  |  |  |  |
| 36. | -.08 | -.13^*^ | .07 | .14^**^ | -.06 | .19^**^ | .03 | -.10^†^ | .02 | -.07 | .05 | -.13^*^ | -.05 | -.13^*^ | -.07 | .01 | .29^**^ | .10^†^ | .07 | .03 | -.17^**^ | -.07 | -.11^†^ | .05 | -.07 | -.13^*^ | .05 | .06 | .34^**^ | .57^**^ | .53^**^ | .57^**^ | .63^**^ | .47^**^ | .63^**^ |  |  |  |  |  |
| 37. | -.12^‡^ | -.12^‡^ | -.00 | .20^**^ | .00 | .16^**^ | .03 | -.18^**^ | .06 | .02 | -.00 | -.16^**^ | -.04 | -.15^**^ | -.06 | .01 | .27^**^ | .09 | .06 | .06 | -.25^**^ | -.08 | -.13^*^ | -.03 | -.11^‡^ | -.17^**^ | .07 | -.01 | .30^**^ | .43^**^ | .64^**^ | .61^**^ | .60^**^ | .46^**^ | .48^**^ | .60^**^ |  |  |  |  |
| 38. | -.11^†^ | -.18^**^ | .07 | .21^**^ | .03 | .18^**^ | .02 | -.16^**^ | -.05 | .00 | .02 | -.12^*^ | -.04 | -.11^†^ | -.05 | .05 | .22^**^ | .06 | .07 | .07 | -.16^**^ | -.08 | -.11^†^ | -.01 | -.05 | -.09^†^ | .07 | -.02 | .27^**^ | .46^**^ | .70^**^ | .65^**^ | .59^**^ | .52^**^ | .48^**^ | .61^**^ | .69^**^ |  |  |  |
| 39. | -.05 | -.18^**^ | .01 | .15^**^ | -.04 | .25^**^ | .10^†^ | -.24^**^ | -.07 | .00 | .02 | -.15^**^ | -.02 | -.15^**^ | -.06 | -.07 | .31^**^ | .09 | .07 | .09^†^ | -.24^**^ | -.11^†^ | -.12^‡^ | -.01 | -.02 | -.14^*^ | .12^‡^ | .01 | .28^**^ | .46^**^ | .63^**^ | .76^**^ | .65^**^ | .54^**^ | .57^**^ | .63^**^ | .67^**^ | .72^**^ |  |  |
| 40. | -.05 | -.26^**^ | .08 | .24^**^ | -.07 | .22^**^ | .00 | -.14^*^ | -.01 | -.00 | .04 | -.16^**^ | -.05 | -.19^**^ | -.03 | -.04 | .29^**^ | .10^†^ | .05 | .07 | -.22^**^ | -.10^†^ | -.17^**^ | -.01 | -.06 | -.12^‡^ | .09^†^ | .03 | .22^**^ | .43^**^ | .53^**^ | .62^**^ | .76^**^ | .47^**^ | .46^**^ | .63^**^ | .62^**^ | .62^**^ | .70^**^ |  |
| 41. | -.06 | -.05 | .03 | .08 | -.03 | .20^**^ | .03 | -.10^†^ | -.05 | -.07 | .07 | .02 | .01 | .01 | .03 | .00 | .12^‡^ | .12^‡^ | .03 | -.01 | -.10^†^ | .02 | -.02 | -.02 | -.03 | -.05 | .09^†^ | .08 | .26^**^ | .40^**^ | .38^**^ | .41^**^ | .39^**^ | .57^**^ | .43^**^ | .44^**^ | .38^**^ | .42^**^ | .45^**^ | .39^**^ |

*Note.* Statistics in this table describe parent responses prior to multiple imputation. 1 = Age; 2 = Gender Identity; 3 = Household Income; 4 = Financial Well-being; 5 = Employment (Ref: Not Working); 6 = Education (Ref: Less than High School); 7 = Married; 8 = White; 9 = English as Home Language; 10 = Number of Children; 11 = First-time Parent; 12 = Program Coach Access Program Preference; 13 = Web-based Access Program Preference; 14 = Teletherapy Access Program Preference; 15 = App-based Access Program Preference; 16 = Program Length Preference; 17 = Peer-driven Program Structure Preference; 18 = Self-directed Program Structure Preference; 19 = Asynchronous Program Structure Preference; 20 = Tailored Content Program Structure Preference; 21 = Psychologist Coaches Preference; 22 = Medical Doctor Coaches Preference; 23 = Social Worker Coaches Preference; 24 = Peer Coaches Preference; 25 = Community Coaches Preference; 26 = Audio-Visual Content Delivery Preference; 27 = Audio-only Content Delivery Preference; 28 = Barriers to Program Access; 29 = Shared Peer Age Identity Preference; 30 = Shared Peer Gender Identity Preference; 31 = Shared Peer Cultural Identity Preference; 32 = Shared Peer Ethnic Identity Preference; 33 = Shared Peer Sexual Orientation Preference; 34 = Shared Peer Education Preference; 35 = Shared Coach Age Identity Preference; 36 = Shared Coach Gender Identity Preference; 37 = Shared Coach Religion Preference; 38 = Shared Coach Cultural Identity Preference; 39 = Shared Coach Ethnic Identity Preference; 40 = Shared Coach Sexual Orientation Preference; 41 = Shared Coach Education Preference.

^†^ < .05. ^‡^ < .01. ^*^ < .005. ^**^ < .001.

*Parent eHealth Program Preferences*

| Variable | *n* | *M* | *SD* | Min | Max |
| --- | --- | --- | --- | --- | --- |
| **Program Features Preference (RQ1) (# options selected)** | - | - | - | - | - |
| Web-based Access Program Preferences | 593 | 0.97 | 0.92 | 0 | 3 |
| App-based Access Program Preferences | 593 | 0.72 | 0.91 | 0 | 3 |
| Program Coach Access Program Preference | 593 | 0.70 | 1.01 | 0 | 3 |
| Teletherapy Access Program Preferences | 593 | 0.26 | 0.54 | 0 | 2 |
| **Program Length Preference (RQ2)** | **484** | **3.09** | **1.55** | **1** | **6** |
| < 1 week (1) | 71 | 14.7% |  |  |  |
| 2-4 weeks (2) | 132 | 27.3% |  |  |  |
| 1-2 months (3) | 120 | 24.8% |  |  |  |
| 3-5 months (4) | 62 | 12.8% |  |  |  |
| 6-12 months (5) | 41 | 8.5% |  |  |  |
| > 1 year (6) | 58 | 12.0% |  |  |  |
| **Program Structure Preference (RQ3)** | - | - | - | - | - |
| Self-directed Program Structure Preference | 520 | 54.75 | 21.53 | 0.0 | 100.0 |
| Peer-driven Program Structure Preference | 517 | 43.69 | 23.00 | 0.0 | 96.0 |
| Asynchronous Program Structure Preference | 506 | 52.76 | 22.54 | 0.0 | 100.0 |
| Tailored Content Program Structure Preference | 499 | - | - | - | - |
| Tailored information for your individual needs | 312 | 62.5% |  |  |  |
| A library of information that you can individually access | 187 | 37.5% |  |  |  |
| **Program Coach Credentials Preference (RQ4)** | - | - | - | - | - |
| Psychologist Coaches Preference | 305 | 51.4% |  |  |  |
| Medical Doctor Coaches Preference | 240 | 40.5% |  |  |  |
| Social Worker Coaches Preference | 201 | 33.9% |  |  |  |
| Community Coaches Preference | 160 | 26.4% |  |  |  |
| Peer Coaches Preference | 134 | 22.6% |  |  |  |
| **Audio-Visual Content Delivery Preference (RQ5)** | **285** | **48.1%** |  |  |  |
| **Barriers to Program Access (RQ6)** | **593** | **1.59** | **1.48** | **0** | **8** |
| **Importance of Shared Peer Identity (RQ7)**  **(1 = “Very unimportant”, 5 = “Very important”)** | - | - | - | - | - |
| Importance of Shared Peer Age Identity | 528 | 3.62 | 1.48 | 1 | 5 |
| Importance of Shared Peer Gender Identity | 530 | 3.49 | 1.26 | 1 | 5 |
| Importance of Shared Peer Cultural Identity | 528 | 3.33 | 1.28 | 1 | 5 |
| Importance of Shared Peer Ethnic Identity | 527 | 3.07 | 1.30 | 1 | 5 |
| Importance of Shared Peer Sexual Orientation Identity | 529 | 3.11 | 1.40 | 1 | 5 |
| Importance of Shared Peer Education Identity | 531 | 3.45 | 1.30 | 1 | 5 |
| **Coach Shared Identity Preference (RQ8)** | - | - | - | - | - |
| Importance of Shared Coach Age Identity | 524 | 3.39 | 1.25 | 1 | 5 |
| Importance of Shared Coach Gender Identity | 529 | 3.27 | 1.31 | 1 | 5 |
| Importance of Shared Coach Religion Identity | 527 | 3.03 | 1.37 | 1 | 5 |
| Importance of Shared Coach Cultural Identity | 530 | 3.21 | 1.33 | 1 | 5 |
| Importance of Shared Coach Ethnic Identity | 527 | 3.04 | 1.33 | 1 | 5 |
| Importance of Shared Coach Sexual Orientation Identity | 530 | 3.07 | 1.41 | 1 | 5 |
| Importance of Shared Coach Education Identity | 530 | 3.68 | 1.27 | 1 | 5 |

*Note.* Statistics in this table describe parent responses prior to multiple imputation. All percentages reflect valid percentages (i.e., the proportion of respondents who endorsed a given item). Those who did not respond are removed from the denominator.

**Sociodemographic Predictors of Parent eHealth Program Preferences – Models Using Raw Dataset**

*Negative Binomial Regression Model Summary for Sociodemographic Predictors of Program Feature Preferences (RQ 1)*

| Outcome | (a) Program Coach Access | | | (b) Web-based Access | | | (c) Teletherapy Access | | | (d) App-based Access | | |
| --- | --- | --- | --- | --- | --- | --- | --- | --- | --- | --- | --- | --- |
|  | Exp(*B*) | 95% CI | *p* | Exp(*B*) | 95% CI | *p* | Exp(*B*) | 95% CI | *p* | Exp(*B*) | 95% CI | *p* |
| Intercept | 0.44 | [0.16, 1.15] | .094 | 1.19 | [0.71, 2.01] | .510 | 0.07 | [0.01, 0.57] | .013 | 0.60 | [0.31, 1.18] | .140 |
| Gender Identity | 1.33 | [1.01, 1.74] | .042 | 0.93 | [0.78, 1.12] | .444 | 1.25 | [0.85, 1.85] | .255 | 1.22 | [0.98, 1.52] | .081 |
| Household Income | 0.99 | [0.92, 1.06] | .792 | 1.06 | [1.01, 1.11] | .019 | 1.00 | [0.91, 1.11] | .942 | 1.02 | [0.97, 1.08] | .464 |
| Financial Well-being | 0.77 | [0.67, 0.88] | <.001* | 0.90 | [0.82, 0.99] | .028 | 0.78 | [0.65, 0.94] | .009 | 0.94 | [0.84, 1.05] | .262 |
| Education (Ref:  < High school) | - | - | - | - | - | - | - | - | - | - | - | - |
| Graduate degree | 2.95 | [1.14, 7.65] | .026 | 0.81 | [0.50, 1.30] | .381 | 6.73 | [0.88, 51.6] | .067 | 0.87 | [0.46, 1.62] | .654 |
| Bachelor’s | 2.68 | [1.07, 6.74] | .036 | 0.74 | [0.47, 1.16] | .188 | 5.32 | [0.71, 39.9] | .104 | 0.86 | [0.47, 1.55] | .608 |
| College | 2.25 | [0.89, 5.67] | .085 | 0.64 | [0.40, 1.02] | .059 | 5.80 | [0.78, 43.3] | .087 | 0.85 | [0.47, 1.54] | .586 |
| High school | 2.54 | [1.01, 6.41] | .048 | 0.68 | [0.42, 1.08] | .100 | 4.94 | [0.66, 37.1] | .120 | 1.08 | [0.60, 1.97] | .792 |
| Married | 0.98 | [0.72, 1.33] | .875 | 1.30 | [1.02, 1.66] | .033 | 0.92 | [0.60, 1.41] | .714 | 1.38 | [1.04, 1.82] | .026 |
| White | 1.37 | [1.07, 1.76] | .014 | 1.07 | [0.09, 1.27] | .472 | 1.48 | [1.03, 2.13] | .034 | 1.03 | [0.83, 1.26] | .802 |

*Note.* *^*^* = *p*-value less than the Benjamini-Hochberg critical *p*-value for this effect. “Gender Identity” is a variable dichotomized ‘woman’ (1) or ‘man’ (0). “Married” and “White” are all variables dichotomized ‘Yes’ (1) or ‘No’ (0).

*Linear Regression Model Summary for Sociodemographic Predictors of Program Length Preferences (RQ 2)*

| Outcome | Program Length | | | |
| --- | --- | --- | --- | --- |
|  | *B* | β | *t* | *p* |
| Intercept | 3.37 |  | 11.34 | < .001 |
| Household Income | 0.07 | .10 | 1.95 | .052 |
| Financial Well-being | -0.20 | -.13 | -2.77 | .006* |
| Married | -0.64 | -.16 | -3.52 | <.001* |
| White | 0.53 | .17 | 3.79 | <.001* |
| Number of Children | 0.13 | .07 | 1.64 | .102 |

*Note.* *^*^* = *p*-value less than the Benjamini-Hochberg critical *p*-value for this effect. “Married” and “White,” are variables dichotomized ‘Yes’ (1) or ‘No’ (0).

*Linear Regression Model Summary for Sociodemographic Predictors of Program Structure Preferences (RQ 3)*

| Outcome | (a) Peer- (vs. Expert-driven) | | | | (b) Self-directed (vs. Structured) | | | | (c) Asynchronous (vs. Sync.) | | | | (d) Tailored (vs. Broad) Content | | |
| --- | --- | --- | --- | --- | --- | --- | --- | --- | --- | --- | --- | --- | --- | --- | --- |
|  | *B* | β | *t* | *p* | *B* | β | *t* | *p* | *B* | β | *t* | *p* | Exp(*B*) | 95% CI | *p* |
| Intercept | 52.43 |  | 6.85 | < .001 | 44.05 |  | 6.13 | < .001 | 55.14 |  | 7.22 | < .001 | 0.91 |  | .889 |
| Age | -0.15 | -.05 | -1.00 | .318 | 0.11 | .04 | 0.78 | .435 | -0.06 | -.02 | -0.38 | .701 | 0.98 | [0.96, 1.01] | .238 |
| Gender Identity | -6.08 | -.13 | -2.66 | .008* | 0.44 | .01 | 0.20 | .839 | -0.97 | -.02 | -0.42 | .674 | 0.84 | [0.55, 1.28] | .427 |
| Household Income | -2.15 | -.19 | -3.81 | <.001* | -0.22 | -.02 | -0.41 | .682 | 0.22 | .02 | 0.39 | .697 | 1.04 | [0.94, 1.15] | .468 |
| Financial Well-being | 2.15 | .10 | 1.85 | .065 | 1.40 | .07 | 1.29 | .198 | 0.85 | .04 | 0.73 | .469 | 0.97 | [0.78, 1.21] | .805 |
| Employment (Ref: Not working) | - | - | - | - | - | - | - | - | - | - | - | - | - | - | - |
| Stay-at-home | 4.31 | .07 | 1.03 | .303 | -3.35 | -.06 | -0.86 | .392 | -4.67 | -.08 | -1.10 | .272 | 1.12 | [0.51, 2.48] | .775 |
| Working | 3.67 | .07 | 0.99 | .325 | 5.80 | .12 | 1.66 | .099 | -3.46 | -.07 | -0.92 | .359 | 1.28 | [0.64, 2.57] | .485 |
| Employment (Ref: Working) | - | - | - | - | - | - | - | - | - | - | - | - | - | - | - |
| Stay-at-home | 0.69 | .01 | 0.22 | .826 | -9.03 | -.15 | -3.08 | .002* | -1.04 | -.02 | -0.33 | .743 | 0.87 | [0.48, 1.58] | .645 |
| Not working | -3.62 | -.05 | -0.96 | .337 | -5.52 | -.08 | -1.56 | .120 | 4.40 | .06 | 1.15 | .250 | 0.74 | [0.37, 1.51] | .413 |

*Note.* *^*^* = *p*-value less than the Benjamini-Hochberg critical *p*-value for this effect. “Gender Identity” is a variable dichotomized ‘woman’ (1) or ‘man’ (0).

*Binary Logistic Regression Model Summary for Sociodemographic Predictors of Program Coach Credential Preferences (RQ 4)*

| Outcome | (a) Psychologist Coaches | | | (b) Medical Doctor Coaches | | | (c) Social Worker Coaches | | |
| --- | --- | --- | --- | --- | --- | --- | --- | --- | --- |
|  | Exp(*B*) | 95% CI | *p* | Exp(*B*) | 95% CI | *p* | Exp(*B*) | 95% CI | *p* |
| Intercept | 0.91 |  | .850 | 0.33 |  | .023 | 0.44 |  | .101 |
| Gender Identity | 1.68 | [1.14, 2.47] | .009 | 1.19 | [0.81, 1.75] | .373 | 1.49 | [0.99, 2.23] | .055 |
| Household Income | 1.13 | [1.02, 1.25] | .015 | 1.16 | [1.06, 1.28] | .002* | 1.14 | [1.03, 1.26] | .010 |
| Financial Well-being | 0.69 | [0.57, 0.85] | <.001* | 0.98 | [0.81, 1.19] | .848 | 0.77 | [0.63, 0.95] | .013 |
| Employment (Ref: Not working) | - | - | - | - | - | - | - | - | - |
| Stay-at-home | 0.55 | [0.27, 1.12] | .098 | 1.11 | [0.55, 2.26] | .774 | 0.42 | [0.20, 0.89] | .024 |
| Working | 0.90 | [0.48, 1.69] | .739 | 0.95 | [0.51, 1.78] | .877 | 0.90 | [0.48, 1.68] | .737 |
| White | 1.94 | [1.36, 2.78] | <.001* | 1.38 | [0.97, 1.97] | .077 | 1.24 | [0.86, 1.81] | .255 |
| English as Home Language | 1.21 | [0.74, 1.97] | .442 | 0.90 | [0.56, 1.46] | .666 | 1.56 | [0.91, 2.68] | .108 |
| First-time Parent | 1.16 | [0.82, 1.64] | .403 | 0.92 | [0.65, 1.30] | .637 | 0.69 | [0.48, 0.99] | .041 |

*Note.* *^*^* = *p*-value less than the Benjamini-Hochberg critical *p*-value for this effect. “Gender Identity” is a variable dichotomized ‘woman’ (1) or ‘man’ (0). “White,” “English as Home Language,” and “First-time Parent” are all variables dichotomized ‘Yes’ (1) or ‘No’ (0).

*Binary Logistic Regression Model Summary for Sociodemographic Predictors of Program Coach Credential Preferences (RQ 4)*

| Outcome | (d) Peer Coaches | | | (d) Community Coaches | | |
| --- | --- | --- | --- | --- | --- | --- |
|  | Exp(*B*) | 95% CI | *p* | Exp(*B*) | 95% CI | *p* |
| Intercept | 0.43 |  | .127 | 0.61 |  | .335 |
| Gender Identity | 0.89 | [0.57, 1.39] | .605 | 1.00 | [0.66, 1.53] | .987 |
| Household Income | 0.95 | [0.85, 1.07] | .398 | 0.95 | [0.86, 1.06] | .371 |
| Financial Well-being | 1.04 | [0.84, 1.30] | .716 | 1.05 | [0.85, 1.29] | .642 |
| Employment (Ref: Not working) | - | - | - | - | - | - |
| Stay-at-home | 1.02 | [0.47, 2.21] | .971 | 0.52 | [0.25, 1.07] | .074 |
| Working | 0.76 | [0.38, 1.51] | .434 | 0.50 | [0.27, 0.93] | .030 |
| White | 0.69 | [0.46, 1.04] | .074 | 1.08 | [0.73, 1.60] | .688 |
| English as Home Language | 1.28 | [0.72, 2.28] | .394 | 1.10 | [0.64, 1.88] | .729 |
| First-time Parent | 0.99 | [0.67, 1.48] | .978 | 1.17 | [0.81, 1.71] | .401 |

*Note.* *^*^* = *p*-value less than the Benjamini-Hochberg critical *p*-value for this effect. “Gender Identity” is a variable dichotomized ‘woman’ (1) or ‘man’ (0). “White,” “English as Home Language,” and “First-time Parent” are all variables dichotomized ‘Yes’ (1) or ‘No’ (0).

*Binary Logistic Regression Model Summary for Sociodemographic Predictors of Audio-Visual Content Delivery Preferences (RQ 5)*

| Outcome | (a) Audio-Visual Content | | | (b) Audio-only Content | | |
| --- | --- | --- | --- | --- | --- | --- |
|  | Exp(*B*) | 95% CI | *p* | Exp(*B*) | 95% CI | *p* |
| Intercept | 1.46 |  | .240 | 0.05 |  | < .001 |
| Household Income | 1.11 | [1.00, 1.22] | .042 | 1.06 | [0.94, 1.20] | .314 |
| Financial Well-being | 0.70 | [0.58, 0.84] | <.001* | 1.30 | [1.03, 1.63] | .028 |
| Education | 1.03 | [0.87, 1.22] | .724 | 1.01 | [0.89, 1.37] | .386 |
| Married | 1.19 | [0.76, 1.84] | .450 | 1.37 | [0.74, 2.56] | .320 |

*Note.* *^*^* = *p*-value less than the Benjamini-Hochberg critical *p*-value for this effect. “Married” is a variable dichotomized ‘Yes’ (1) or ‘No’ (0).

*Negative Binomial Regression Model Summary for Sociodemographic Predictors of Barriers to Program Access (RQ 6)*

| Outcome | Barriers to Program Access | | |
| --- | --- | --- | --- |
|  | Exp(*B*) | 95% CI | *p* |
| Intercept | 1.93 | [0.93, 4.02] | 077 |
| Age | 0.98 | [0.97, 1.00] | .004* |
| Gender Identity | 0.91 | [0.77, 1.08] | .294 |
| Household Income | 0.98 | [0.94, 1.03] | .451 |
| Financial Well-being | 0.94 | [0.86, 1.02] | .141 |
| Employment (Ref: Not working) | - | - | - |
| Stay-at-home | 0.76 | [0.56, 1.03] | .078 |
| Working | 0.90 | [0.69, 1.17] | .415 |
| Employment (Ref: Working) | - | - | - |
| Stay-at-home | 0.85 | [0.67, 1.08] | .172 |
| Not working | 1.12 | [0.86, 1.45] | .415 |
| Education (Ref: Less than High School) | - | - | - |
| Graduate or Professional Degree | 2.10 | [1.20, 3.68] | .010 |
| Bachelor’s Degree | 2.11 | [1.23, 3.63] | .007* |
| College/Technical School | 1.73 | [1.00, 2.99] | .050 |
| High School | 2.13 | [1.23, 3.67] | .007* |
| White | 1.27 | [1.09, 1.48] | .002* |

*Note.* *^*^* = *p*-value less than the Benjamini-Hochberg critical *p*-value for this effect. “Gender Identity” is a variable dichotomized ‘woman’ (1) or ‘man’ (0). “White” is a variable dichotomized ‘Yes’ (1) or ‘No’ (0).

*Linear Regression Model Summary for Sociodemographic Predictors of Preferences for Shared Peer and Coach Identity (RQ 7, 8)*

| Outcome | (a) Shared Peer Age Identity | | | | (b) Shared Peer Gender Identity | | | | (c) Shared Peer Cultural Identity | | | | (d) Shared Peer Ethnic Identity | | | |
| --- | --- | --- | --- | --- | --- | --- | --- | --- | --- | --- | --- | --- | --- | --- | --- | --- |
|  | *B* | β | *t* | *p* | *B* | β | *t* | *p* | *B* | β | *t* | *p* | *B* | β | *t* | *p* |
| Intercept | 3.61 |  | 9.03 | <.001 | 4.12 |  | 9.83 | <.001 | 3.38 |  | 8.11 | <.001 | 2.78 |  | 6.60 | <.001 |
| Age | 0.00 | .00 | -0.02 | .987 | -0.01 | -.06 | -1.15 | .251 | -0.01 | -.06 | -1.19 | .235 | 0.00 | -.02 | -0.44 | .662 |
| Gender Identity | 0.00 | .00 | 0.03 | .980 | -0.29 | -.11 | -2.39 | .017 | -0.44 | -.16 | -3.61 | <.001* | -0.41 | -.15 | -3.38 | <.001* |
| Household Income | 0.01 | .01 | 0.25 | .802 | -0.04 | -.06 | -1.23 | .220 | -0.01 | -.02 | -0.45 | .650 | -0.06 | -.09 | -1.75 | .082 |
| Financial Well-being | -0.03 | -.02 | -0.46 | .643 | 0.00 | .00 | -0.05 | .957 | 0.15 | .12 | 2.36 | .019 | 0.21 | .16 | 3.32 | <.001* |
| Education | 0.10 | .10 | 1.89 | .060 | 0.14 | .12 | 2.41 | .016 | 0.16 | .14 | 2.85 | .005* | 0.20 | .16 | 3.43 | <.001* |
| Married | -0.19 | -.06 | -1.32 | .188 | -0.13 | -.04 | -0.84 | .402 | -0.01 | .00 | -0.07 | .942 | 0.07 | .02 | 0.44 | .663 |
| White | -0.18 | -.07 | -1.61 | .108 | -0.28 | -.11 | -2.46 | .014 | -0.30 | -.12 | -2.62 | .009 | -0.48 | -.18 | -4.19 | <.001* |
| First-time Parent | 0.15 | .06 | 1.39 | .166 | -0.01 | .00 | -0.04 | .967 | -0.09 | -.04 | -0.80 | .423 | 0.02 | .01 | 0.14 | .893 |

*Note.* *^*^* = *p*-value less than the Benjamini-Hochberg critical *p*-value for this effect. “Gender Identity” is a variable dichotomized ‘woman’ (1) or ‘man’ (0). “Married,” “White,”  and “First-time Parent” are all variables dichotomized ‘Yes’ (1) or ‘No’ (0).

*Linear Regression Model Summary for Sociodemographic Predictors of Preferences for Shared Peer and Coach Identity (RQ 7, 8)*

| Outcome | (e) Shared Peer Sexual Orientn. | | | | (f) Shared Peer Education | | | | (g) Shared Coach Age Identity | | | | (h) Shared Coach Gender Identity | | | |
| --- | --- | --- | --- | --- | --- | --- | --- | --- | --- | --- | --- | --- | --- | --- | --- | --- |
|  | *B* | β | *t* | *p* | *B* | β | *t* | *p* | *B* | β | *t* | *p* | *B* | β | *t* | *p* |
| Intercept | 3.38 |  | 7.39 | <.001 | 3.05 |  | 7.15 | <.001 | 3.47 |  | 8.45 | <.001 | 3.28 |  | 7.48 | <.001 |
| Age | -0.02 | -.09 | -2.06 | .040 | -0.01 | -.04 | -0.91 | .363 | -0.01 | -.06 | -1.30 | .195 | -0.02 | -.08 | -1.72 | .087 |
| Gender Identity | -0.49 | -.17 | -3.73 | <.001* | -0.20 | -.07 | -1.61 | .109 | -0.40 | -.15 | -3.35 | <.001* | -0.32 | -.12 | -2.52 | .012 |
| Household Income | -0.05 | -.07 | -1.46 | .144 | -0.02 | -.04 | -0.72 | .471 | -0.05 | -.07 | -1.46 | .145 | -0.02 | -.03 | -0.67 | .502 |
| Financial Well-being | 0.15 | .11 | 2.15 | .032 | 0.15 | .12 | 2.36 | .019 | 0.08 | .06 | 1.26 | .210 | 0.11 | .08 | 1.65 | .100 |
| Education | 0.25 | .20 | 4.06 | <.001* | 0.22 | .18 | 3.75 | <.001* | 0.17 | .15 | 3.01 | .003* | 0.22 | .18 | 3.65 | <.001* |
| Married | 0.02 | .01 | 0.11 | .911 | -0.01 | .00 | -0.09 | .926 | 0.01 | .00 | 0.09 | .926 | -0.03 | -.01 | -0.17 | .867 |
| White | -0.29 | -.10 | -2.35 | .019 | -0.25 | -.10 | -2.16 | .031 | -0.09 | -.04 | -0.83 | .406 | -0.11 | -.04 | -0.89 | .372 |
| First-time Parent | 0.00 | .00 | 0.01 | .991 | 0.09 | .04 | 0.79 | .428 | 0.26 | .11 | 2.33 | .020 | 0.04 | .02 | 0.33 | .738 |

*Note.* *^*^* = *p*-value less than the Benjamini-Hochberg critical *p*-value for this effect. “Gender Identity” is a variable dichotomized ‘woman’ (1) or ‘man’ (0). “Married,” “White,”  and “First-time Parent” are all variables dichotomized ‘Yes’ (1) or ‘No’ (0).

*Linear Regression Model Summary for Sociodemographic Predictors of Preferences for Shared Peer and Coach Identity (RQ 7, 8)*

| Outcome | (i) Shared Coach Religion | | | | (j) Shared Coach Cultural Identity | | | | (k) Shared Coach Ethnic Identity | | | |
| --- | --- | --- | --- | --- | --- | --- | --- | --- | --- | --- | --- | --- |
|  | *B* | β | *t* | *p* | *B* | β | *t* | *p* | *B* | β | *t* | *p* |
| Intercept | 3.28 |  | 7.36 | <.001 | 3.54 |  | 8.31 | <.001 | 3.03 |  | 7.15 | <.001 |
| Age | -0.02 | -.10 | -2.25 | .025 | -0.02 | -.11 | -2.43 | .016 | -0.01 | -.05 | -1.05 | .295 |
| Gender Identity | -0.29 | -.10 | -2.29 | .022 | -0.43 | -.16 | -3.47 | <.001* | -0.45 | -.16 | -3.70 | <.001* |
| Household Income | -0.08 | -.12 | -2.49 | .013 | -0.02 | -.03 | -0.59 | .557 | -0.09 | -.13 | -2.75 | .006* |
| Financial Well-being | 0.23 | .17 | 3.50 | <.001* | 0.17 | .13 | 2.57 | .010 | 0.13 | .10 | 2.03 | .042 |
| Education | 0.21 | .17 | 3.38 | <.001* | 0.21 | .17 | 3.53 | <.001* | 0.28 | .23 | 4.81 | <.001* |
| Married | 0.04 | .01 | 0.26 | .799 | -0.09 | -.03 | -0.58 | .562 | 0.25 | .07 | 1.67 | .096 |
| White | -0.32 | -.12 | -2.62 | .009* | -0.26 | -.10 | -2.25 | .025 | -0.47 | -.18 | -4.10 | <.001* |
| First-time Parent | -0.16 | -.06 | -1.30 | .194 | -0.09 | -.03 | -0.74 | .461 | -0.07 | -.03 | -0.58 | .564 |

*Note.* *^*^* = *p*-value less than the Benjamini-Hochberg critical *p*-value for this effect. “Gender Identity” is a variable dichotomized ‘woman’ (1) or ‘man’ (0). “Married,” “White,”  and “First-time Parent” are all variables dichotomized ‘Yes’ (1) or ‘No’ (0).

*Linear Regression Model Summary for Sociodemographic Predictors of Preferences for Shared Peer and Coach Identity (RQ 7, 8)*

| Outcome | (l) Shared Coach Sexual Orientn. | | | | (m) Shared Coach Education | | | |
| --- | --- | --- | --- | --- | --- | --- | --- | --- |
|  | *B* | β | *t* | *p* | *B* | β | *t* | *p* |
| Intercept | 2.86 |  | 6.37 | <.001 | 3.56 |  | 8.42 | <.001 |
| Age | -0.01 | -.04 | -0.90 | .369 | -0.01 | -.06 | -1.30 | .196 |
| Gender Identity | -0.64 | -.22 | -4.99 | <.001* | -0.11 | -.04 | -0.90 | .370 |
| Household Income | -0.05 | -.07 | -1.46 | .146 | -0.03 | -.04 | -0.82 | .415 |
| Financial Well-being | 0.23 | .17 | 3.48 | <.001* | 0.05 | .04 | 0.72 | .474 |
| Education | 0.27 | .21 | 4.43 | <.001* | 0.23 | .20 | 3.90 | <.001* |
| Married | -0.19 | -.05 | -1.20 | .231 | 0.00 | .00 | 0.01 | .988 |
| White | -0.19 | -.07 | -1.54 | .124 | -0.11 | -.04 | -0.98 | .329 |
| First-time Parent | -0.02 | -.01 | -0.19 | .854 | 0.09 | .04 | 0.76 | .451 |

*Note.* *^*^* = *p*-value less than the Benjamini-Hochberg critical *p*-value for this effect. “Gender Identity” is a variable dichotomized ‘woman’ (1) or ‘man’ (0). “Married,” “White,”  and “First-time Parent” are all variables dichotomized ‘Yes’ (1) or ‘No’ (0).

**Appendix D: Parent Preferences Questionnaire**

**Instructions:** The following section includes questions related to your preferences for engaging in an eHealth (**online**) resource focused on family mental health. The term family mental health refers to a range of challenges that many families experience, such as 1) mental health problems for parents, 2) mental health problems for children, and 3) family conflict between two caregivers or between a caregiver and a child.

1. **Content through eHealth resources can be delivered in a variety of different ways. How would you want to access content through a program? (Check all that apply)**

- Web-based portal on computer or laptop
- Web-based portal on tablet
- Web-based portal on phone
- Application on computer or laptop
- Application on tablet
- Application on phone
- Text messages from a coach or healthcare provider
- Emails from a coach or healthcare provider
- Teletherapy on videoconferencing platforms (e.g., Zoom, Skype)
- Teletherapy over the phone
- Virtual meetings with a coach on a videoconferencing platform (e.g., Zoom, Skype)
- Telephone calls with a coach or healthcare provider
- Not listed (please specify): ______
- ⊗Prefer not to answer

1. **How long would you want to be enrolled in a program?**

- <1 week
- 2 - 4 weeks
- 1 - 2 months
- 3 - 5 months
- 6 - 12 months
- >1 year
- Prefer not to answer

1. **How long (hours and minutes) would you want to spend engaging in a program?**
2. Each day: ___ hours ___minutes
3. Each week: ___ hours ___minutes
4. Each month: ___ hours ___minutes

- Prefer not to answer

1. **How many reminders/notifications from the program would you want to receive?**
2. Each day: ___
3. Each week: ___
4. Each month: ___

- Prefer not to answer

1. **If you were experiencing family mental health challenges, which of the following eHealth features would you be interested in accessing…**
2. **immediately? (Check all that apply)**

- Content, such as readings and videos, developed by experts (i.e., doctors, psychologists, social workers)
- Content, such as readings and videos, developed by peers (i.e., fellow parents, community coaches)
- Supportive online forum
- Virtual peer coaching
- Virtual individual therapy
- Virtual couple therapy
- Virtual family therapy
- Virtual support group
- Not listed (please specify): _____
- ⊗None
- ⊗Prefer not to answer

1. **in six months, if the challenges persisted? (Check all that apply)**

- Content, such as readings and videos, developed by experts (i.e., doctors, psychologists, social workers)
- Content, such as readings and videos, developed by peers (i.e., fellow parents, community coaches)
- Supportive online forum
- Virtual peer coaching
- Virtual individual therapy
- Virtual couple therapy
- Virtual family therapy
- Virtual support group
- Not listed (please specify): _____
- ⊗None
- ⊗Prefer not to answer

*Branching logic: If participant did not check “none” or “prefer not to answer” on 5a OR 5b*

1. **Which eHealth feature would be your #1 preferred way to address a family mental health challenge?**

- Content, such as readings and videos, developed by experts (i.e., doctors, psychologists, social workers)
- Content, such as readings and videos, developed by peers (i.e., fellow parents, community coaches)
- Supportive online forum
- Virtual peer coaching
- Virtual individual therapy
- Virtual couple therapy
- Virtual family therapy
- Virtual support group
- Not listed (please specify): _____
- None
- Prefer not to answer

*Descriptive text: eHealth programs can be structured in different ways. The following set of questions ask about your preferences in program structure.*

1. **Self-directed programs allow participants to engage with content of their choosing while structured programs have participants engage with specified content. Would you want a program to be self-directed or structured?**

|  | Self-directed | Mix of Both | Structured |
| --- | --- | --- | --- |

|  | 0 | 25 | 50 | 75 | 100 |
| --- | --- | --- | --- | --- | --- |

|  | 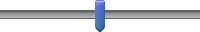 |
| --- | --- |

1. Prefer not to answer

1. **Asynchronous programs allow participants to engage with the content on their own convenience while synchronous programs have participants engage with the content at a regular time each week. Would you want a program to be synchronous or asynchronous?**

|  | Synchronous | Mix of Both | Asynchronous |
| --- | --- | --- | --- |

|  | 0 | 25 | 50 | 75 | 100 |
| --- | --- | --- | --- | --- | --- |

| Synchronous or asynchronous () | 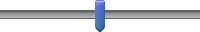 |
| --- | --- |

1. Prefer not to answer

1. **Expert-driven programs are led by experts in family mental health, such as psychologists, social workers, and medical doctors. Peer-driven programs are led by fellow parents and community coaches. Would you like a program to be expert- or peer-driven?**

|  | Expert-driven | Mix of Both | Peer-driven |
| --- | --- | --- | --- |

|  | 0 | 25 | 50 | 75 | 100 |
| --- | --- | --- | --- | --- | --- |

|  | 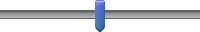 |
| --- | --- |

1. Prefer not to answer

1. **Would you want a program to provide**

- Tailored information for your individual needs
- A library of information that you can individually access
- Prefer not to answer

1. **Would you want to receive coaching from: (Check all that apply)**

- Peers
- Community coaches
- Medical doctors
- Psychologists
- Social workers
- Not listed (please specify): ____
- ⊗Prefer not to answer

*Branching logic: If participant did not check prefer not to answer option on the previous question*

1. **Who would be your #1 choice to receive coaching from:**

- Peers
- Community coaches
- Medical doctors
- Psychologists
- Social workers
- Not listed (please specify): ____
- Prefer not to answer

1. **How important would it be to have fellow parents on the platform with a shared identity in the following domains:**

|  | Very unimportant | Somewhat Unimportant | Neither unimportant or important | Somewhat important | Very important | Prefer not to answer |
| --- | --- | --- | --- | --- | --- | --- |
| Age |  |  |  |  |  |  |
| Gender |  |  |  |  |  |  |
| Religion |  |  |  |  |  |  |
| Culture |  |  |  |  |  |  |
| Ethnicity |  |  |  |  |  |  |
| Sexual Orientation |  |  |  |  |  |  |
| Education Level |  |  |  |  |  |  |

1. **How important would it be to have a coach with a shared identity in the following domains:**

|  | Very unimportant | Somewhat Unimportant | Neither unimportant or important | Somewhat important | Very important | Prefer not to answer |
| --- | --- | --- | --- | --- | --- | --- |
| Age |  |  |  |  |  |  |
| Gender |  |  |  |  |  |  |
| Religion |  |  |  |  |  |  |
| Culture |  |  |  |  |  |  |
| Ethnicity |  |  |  |  |  |  |
| Sexual Orientation |  |  |  |  |  |  |
| Education Level |  |  |  |  |  |  |

1. **Is there anyone in your household that you would want to participate in a parenting program alongside you?**

- Yes, my partner
- Yes, another caregiver
- Yes, someone else (please specify): _______________
- No
- I don't know
- Prefer not to answer

*Branching logic: If participant responded with “yes, my partner” OR “yes, another caregiver” OR “yes, someone else” to previous question*

1. **How would you want this person to participate? (Check all that apply)**

- Interact with content separately
- Interact with content with you
- Contribute to group discussions separately
- Contribute to group discussions with you
- Complete home practice activities separately
- Complete home practice activities with you
- ⊗Prefer not to answer

1. **How much would you like to have access to the following features:**

|  | Dislike a great deal | Dislike somewhat | Neither like nor dislike | Like somewhat | Like a great deal | Prefer not to answer |
| --- | --- | --- | --- | --- | --- | --- |
| Home practice activities |  |  |  |  |  |  |
| Daily reminders |  |  |  |  |  |  |
| Weekly reminders |  |  |  |  |  |  |
| Progress and symptom monitoring |  |  |  |  |  |  |
| Connecting with parents through a forum |  |  |  |  |  |  |

1. **How would you want content to be presented? (Check all that apply)**

- Audio-only materials, such as podcasts and audio testimonials
- Visual-only materials, such as articles and blog posts
- Audio-visual materials, such as video roleplays and presentations
- ⊗Prefer not to answer

*Branching logic: if participant checked audio-only materials option on question 17*

1. **For audio-only materials (podcasts, audio testimonials)...**
2. **How long would you want to listen to family mental health content in a day (in minutes)?**

|  | 0 |  |  |  | 60+ |
| --- | --- | --- | --- | --- | --- |

|  | 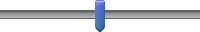 |
| --- | --- |

1. Prefer not to answer

1. **If the audio clips were 3-minutes long, how many would you want to listen to in a week?**

|  | 0 |  | 3 |  | 6+ |
| --- | --- | --- | --- | --- | --- |

|  | 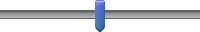 |
| --- | --- |

1. Prefer not to answer

*Branching logic: if participant checked visual-only materials option on question 17*

1. **For visual-only materials (articles, blog posts, infographics)...**
2. **How long would you want to read family mental health content in a day (in minutes)?**

|  | 0 |  |  |  | 60+ |
| --- | --- | --- | --- | --- | --- |

|  | 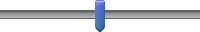 |
| --- | --- |

1. Prefer not to answer

1. **If each material was 1-page long, how many pages of family mental health content would you want to read in a week?**

|  | 0 |  | 3 |  | 6+ |
| --- | --- | --- | --- | --- | --- |

|  | 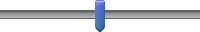 |
| --- | --- |

1. Prefer not to answer

*Branching logic: if participant checked audio-visual materials option on question 17*

1. **For audio-visual materials (such as video role-plays, video presentations, video testimonials)...**
2. **How long would you want to watch family mental health content at in a day (in minutes)?**

|  | 0 |  |  |  | 60+ |
| --- | --- | --- | --- | --- | --- |

|  | 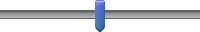 |
| --- | --- |

1. Prefer not to answer

1. **If each video was 3-minutes long, how many videos of family mental health content would you want to watch each week?**

|  | 0 |  | 3 |  | 6+ |
| --- | --- | --- | --- | --- | --- |

|  | 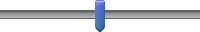 |
| --- | --- |

1. Prefer not to answer

1. **Have you used any of the following online mental health or parenting resources? (Check all that apply)**

- MoodMission
- HeadSpace
- Calm
- SolidStarts
- TalkSpace
- MindShift
- Bundoo
- Not listed (please specify): ______________________
- ⊗None
- ⊗Prefer not to answer

1. **What did you like about the resource(s) you used? If you would prefer not to answer this question, please type 888 into the text box**

_________

1. **What did you dislike about the resource(s) you used? If you would prefer not to answer this question, please type 888 into the text box**

_________

1. **Have you used any of the following social media platforms for mental health or parenting support? (Check all that apply)**

- Facebook
- Youtube
- Instagram
- Reddit
- Twitter
- TikTok
- Snapchat
- Quora
- Not listed (please specify): ______________________________________
- ⊗None
- ⊗Prefer not to answer

*Branching logic for questions 26-28: if participant did not check “none” or “prefer not to answer” for question 25.*

1. **What did you like about the social media platform(s) you used for support? If you would prefer not to answer this question, please type 888 into the text box**

___________________________________________________

1. **What did you dislike about the social media platform(s) you used for support? If you would prefer not to answer this question, please type 888 into the text box**

____________________________________________________

1. **How useful did you find the mental health and/or parenting support you received from social media?**

|  | Not Useful | Neutral | Very Useful |
| --- | --- | --- | --- |

|  | | 0 | 10 | 20 | 30 | 40 | 50 | 60 | 70 | 80 | 90 | 100 |  |
| --- | --- | --- | --- | --- | --- | --- | --- | --- | --- | --- | --- | --- | --- |
|  | 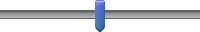 | | | | | | | | | | | | |

1. Prefer not to answer

1. **Which of the following factors could you foresee being a potential barrier to being involved in an online mental health or parenting program? (Check all that apply)**

- Wi-Fi capabilities (e.g., slow internet)
- Limited access to electronic device within household (e.g., devices being used by other members of the household)
- Functional capabilities of electronic device (e.g., unable to download applications)
- Technology literacy (e.g., lacking knowledge in navigating online platforms)
- Lack of time (e.g., limits on the amount of time to spend on program)
- Lack of space (e.g., no private space to participate in program within the home)
- Lack of childcare (e.g., no one to look after child while participating in program)
- Lack of interest (e.g., losing interest in the program)
- Not listed (please specify): _____________________________________
- ⊗None
- ⊗Prefer not to answer

1. **What would help facilitate your involvement in an online mental health or parenting program? If you would prefer not to answer this question, please type 888 into the text box**

__________________________________________________
